# A field study of the impacts of workplace diversity on the recruitment of minority group members

**DOI:** 10.1038/s41562-023-01731-5

**Published:** 2023-10-30

**Authors:** Aaron D. Nichols, Jordan Axt, Evelyn Gosnell, Dan Ariely

**Affiliations:** 1 Department of Marketing, Questrom School of Business, Boston University, Boston, MA USA; 2 Department of Psychology, McGill University, Montreal, Quebec Canada; 3 Irrational Labs, San Francisco, CA USA; 4 Center for Advanced Hindsight, Duke University, Durham, NC USA

**Keywords:** Social sciences, Business and management, Psychology, Human behaviour, Business and management

## Abstract

Increasing workplace diversity is a common goal. Given research showing that minority applicants anticipate better treatment in diverse workplaces, we ran a field experiment (*N* = 1,585 applicants, *N* = 31,928 website visitors) exploring how subtle organizational diversity cues affected applicant behaviour. Potential applicants viewed a company with varying levels of racial/ethnic or gender diversity. There was little evidence that racial/ethnic or gender diversity impacted the demographic composition or quality of the applicant pool. However, fewer applications were submitted to organizations with one form of diversity (that is, racial/ethnic or gender diversity), and more applications were submitted to organizations with only white men employees or employees diverse in race/ethnicity and gender. Finally, exploratory analyses found that female applicants were rated as more qualified than male applicants. Presenting a more diverse workforce does not guarantee more minority applicants, and organizations seeking to recruit minority applicants may need stronger displays of commitments to diversity.

## Abstract (web summary)

A Registered Report field study by Nichols et al. finds little evidence that images of organizational diversity change the volume or quality of minority applicants. Stronger commitments to diversity may be needed to increase recruitment of minority applicants.

## Main

Increasing workplace diversity is a popular but elusive goal for many employers. Over the past several decades, equal opportunity initiatives and new policies in business have encouraged employers to make concerted efforts to hire diverse personnel^1^. However, despite numerous regulations and new efforts in diversity recruitment, many organizations struggle to hire employees from minority backgrounds^2,3^, with even well-intentioned initiatives to improve diversity backfiring or creating adverse reactions among minority employees^4,5^. This lack of workplace diversity is particularly problematic within white-collar industries, where Black men and women are still underrepresented^6^; as recently as 2015, there were more Fortune 500 CEOs named John or David than there were women^7,8^. Why are these organizations unable to increase the diversity of their workforces? How can organizations encourage applications from talented employees that come from all walks of life? In this work, we investigate how workplace diversity cues affect the quality and quantity of job applications from both minority and majority groups.

One difficulty that organizations may have in creating a diverse workforce is in communicating to applicants that their organization is one where members of minority groups can anticipate being treated with respect and fairness. According to social identity theory^9^, individuals attend to important characteristics (that is, identities like age, gender and race) to create identities and to differentiate themselves and others. Individuals often have multiple social identities^10–12^, and the salience of these identities can influence behaviour. For instance, individuals tend to seek out environments that affirm their identity^13^, especially when they have previously experienced discrimination^14^.

Past work has shown that social identity is a key factor in determining individuals’ organizational commitment and comfort^13,15,16^. Not only do employees use their organizational affiliations to form their social identity, they also experience more positive outcomes when these organizations reflect their existing social identity and values. For instance, racial minorities working in organizations with greater perceived commitment to diversity expressed a lower desire to leave their current positions^17^. Conversely, in contexts where minority applicants anticipate discrimination, they are more likely to alter their presentation as a potential employee and engage in ‘resume whitening’, such as through removing experience in organizations that could signal racial identity (for example, the National Society for Black Engineers)^18^. To the extent that such positive or negative outcomes can be anticipated, applicants from minority groups should be more attracted to organizations where they are more similar to other employees.

A straightforward implication of this prior work is that organizations could communicate to applicants from minority groups that they would be entering an inclusive environment through front-facing materials, such as company websites, that highlight existing organizational diversity. Indeed, prior work has found that people readily use such materials to form impressions of an organization. For instance, it was observed that when company-brochure images depicted a high level of minority representation, Black professionals were more trusting of the company regardless of the company’s stated diversity philosophy^19^. Furthermore, Black professionals’ increase in organizational trust was mediated by a decrease in the activation of social identity contingencies—that is, concerns about being marginalized because of one’s racial identity. This work highlights how workplace diversity cues are important to workers from underrepresented groups, as these cues can affect trust and social identity contingencies.

Similar findings have emerged in other studies. For example, one lab study found that presenting a fictional departmental website with images that displayed greater diversity in the student population (here, in terms of age) led student participants to think the department placed a greater value on diversity and that they would be treated with more respect^20,21^. Additionally, a field study among ethnic minorities in Belgium found that job advertisements were rated less positively when they stressed the importance of traits (for example, integrity) that minority participants believed to be the topic of negative stereotypes that others held towards their own group^22^. In general, the research indicates that people are sensitive to job materials that subtly indicate a potentially unwelcoming environment.

Together, these studies highlight correlational and experimental evidence that members of minority groups experience more positive outcomes in contexts with greater diversity, and that minority group members will use organizational materials such as websites and job advertisements to infer the likelihood of feeling welcomed. Although this work is certainly informative, the literature has noticeably few field studies that combine the external validity of investigating real-world decisions with experimental manipulations that can identify sources of causality in behaviour. Moreover, while there are several field studies that use experimental approaches to identify how employers evaluate applicants from minority groups^18,23,24^, fewer field studies investigate how applicants use information related to an employer’s treatment of minority group members to make consequential employment decisions.

A field study on organizational diversity cues would be highly useful for two reasons. First, many prior studies finding that members of minority groups experience more positive outcomes in more diverse workplaces are ultimately correlational, thereby leaving open the possibility that other factors are responsible for this relationship (for example, organizations with greater diversity may also be more likely to invest in forms of employee wellness or provide better benefits). Second, the existing experimental evidence showing the impact of diversity cues has needed to rely on secondary outcomes (for example, anticipated respect^20^), all while completed in an environment where the participants know that their responses will be evaluated. Indeed, the literature on workplace diversity cues is limited in scope and external validity. The prior work has relied on outcomes such as self-reported organizational attractiveness^19,22,25–31^ and anticipated trust^19,29^ rather than a more externally valid measure such as willingness to apply to join an organization. In sum, while research indicates that subtle details can induce social identity threats^32–34^, decrease organizational trust and comfort^19,29^, and weaken job appeal^19,22,25–31^, the literature lacks direct evidence that organizational cues can affect actual applicant behaviour in an ecologically valid context.

We aimed to build on existing research and address this prominent gap in the literature by conducting a naturalistic field study testing the effect of diversity cues, conveyed through online images of an organization’s members, on the quality and quantity of applicants from both minority and majority groups. We studied the recruitment of female and non-white applicants in a context where women and people of colour are stigmatized and biases are prominent: technology^35–37^.

**Fig. 1:**
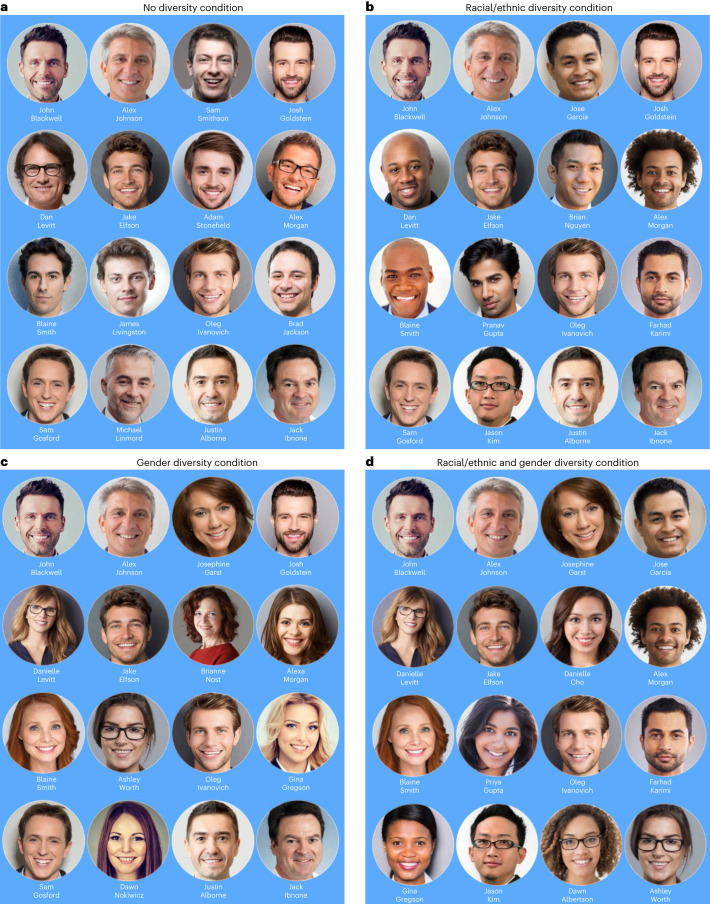
Images used to convey organizational diversity. **a**, The no diversity condition contained only white men. **b**, The racial/ethnic diversity condition contained men that were both white and non-white. **c**, The gender diversity condition contained white men and women. **d**, The racial/ethnic and gender diversity condition contained white and non-white men and women. See the Supplementary Information for the credit line for this figure.

To do so, we created a realistic website for a hypothetical technology startup company with several job openings. Visitors were recruited through online advertising and were randomly assigned to view images of the company’s workforce that were either high or low in gender diversity, racial/ethnic diversity or both. Using a 2 × 2 between-participant experimental design, we manipulated the presence or absence of racial/ethnic and gender diversity (Fig. 1), such that images of the organization’s employees were (1) all white men, (2) white men and white women, (3) white men and non-white men or (4) both white and non-white men and women.

For comparison purposes, we operationalized the all-white male condition as our positive control. We investigated whether this manipulation of organizational diversity cues impacted the behaviour (that is, the decision to apply for a job) of potential applicants. To maximize statistical power and minimize threats to statistical validity, we investigated workplace diversity effects across stigmatized and non-stigmatized groups based on race/ethnicity (non-white versus white) and gender (female versus male). Specifically, we examined how such subtle organizational diversity cues affected the quantity and quality of applications that were submitted from non-white men, non-white women, white men and white women.

In addition to using a realistic and consequential outcome such as the decision to submit a job application, our design allows for tests concerning how members of minority and majority groups respond to different signals of organizational diversity. For one, this study can investigate the extent to which applicant behaviour is impacted by the presence of organizational diversity cues that promote members of other minority groups (for example, how non-white men react to an organization that seems willing to hire women but not racial/ethnic minorities). Second, this work can explore the behaviour of members of majority groups (that is, white men), which might add nuance to prior work that has observed conflicting evidence concerning the degree to which majority group members react to organizational diversity cues^25,27–29,38^.

Taken together, this work can advance understanding on issues concerning employee recruitment and intergroup relations, while also providing an externally valid investigation of organizational diversity cues and their role in the recruitment of minority group members. Below, we review the prior evidence that informs possible study outcomes.

First, one clear prediction from past research is that applicants from minority groups will have more positive evaluations of organizations that employ members who share their same identity, leading to a greater likelihood of submitting a job application. Previous work consistently finds that minority group members respond positively to seeing others with similar identities in organizational materials. In one example, Black female undergraduates were presented with a picture of a fictional school’s science department and then read a profile of a faculty member who was either a Black or white male or female^39^. The study found that participants indicated greater anticipated belonging and trust at the school when presented with a Black faculty member (either male or female). Similarly, Black female participants evaluated a hypothetical technology company more positively when the company’s materials included the profile of a current male or female Black employee^40^. Moreover, Latina women rated a hypothetical STEM company more positively when the company displayed the profile of a current Latino or Latina employee^41^.

These lab studies are complemented by correlational research that finds higher job satisfaction among minority employees working in settings where they share greater similarity with those around them. For instance, employees who were racial/ethnic minorities reported lower levels of perceived discrimination at work when their supervisor shared their demographic background^42^. Conversely, racial minority students working in study groups with greater ethnic dissimilarity to themselves reported lower feelings of belonging and reduced organizational attachment^43^.

To the extent that participants from minority groups can anticipate the positive consequences of working in an organization where other coworkers have the same identity, one hypothesis is that workplace diversity cues will affect participants’ preferences and job application behaviour. Specifically, members of minority groups should be more likely to submit a job application when viewing a company whose members show greater similarity to their own stigmatized identity or identities (Hypotheses 1, 3 and 5 in Supplementary Table 1). In this case, we would anticipate white women applying more to organizations that are shown to have greater gender diversity and non-white men applying more to organizations that are shown to have greater racial/ethnic diversity. A small exception concerns non-white women, who are also expected to apply more to organizations showing greater racial/ethnic (but not gender) diversity, given prior findings of Black and Latina female participants showing greater organizational attractiveness when materials highlighted a female or male employee of the same racial/ethnic identity^40,41^.

However, another series of possible outcomes emerges from more recent work concerning how members of minority groups will be impacted by organizational diversity cues that promote members with differing under-represented identities. While there are relatively straightforward predictions concerning how job applicants from minority groups will evaluate organizations whose members share their own race/ethnicity or gender, there is more mixed evidence on how such applicants will evaluate organizations whose workforces signal a different kind of diversity, one that promotes other under-represented groups (for example, white women viewing an organization that has racial/ethnic but not gender diversity). Some research suggests that the impact of diversity may be rather narrow, as minority group members’ evaluations of organizational diversity are overly reliant on the presence or absence of group members who share their own stigmatized identity. The impact of diversity messaging may also be specific to certain marginalized identities; for example, Black female participants’ evaluation of a hypothetical company was affected by materials promoting racial but not gender diversity^39^.

Indeed, people appear to be biased in their assessments of diversity. For instance, Black and Asian participants gave lower ratings of diversity when a company had minority employees that differed from the participants’ own race; that is, Asian participants thought there was lower diversity at a company whose only racial minority employees were Black than at a company whose only racial minority employees were Asian^44^. Applying these findings to our own design, we might anticipate that perceptions of organizational diversity, a key factor in determining whether members of minority groups will apply for a job, will be impacted by the degree to which applicants see their own racial/ethnic or gender identities reflected in the current employees (for example, white women should view the all-white company that has gender diversity as more diverse, and therefore more appealing, than the all-male company that has racial/ethnic diversity).

At the same time, such results are in contrast with work finding ‘spillover’ biases in diversity judgements. In one example, participants evaluating groups of faces containing equal numbers of men versus women across groups, but differing numbers of racial minority members, provided higher ratings of gender diversity to those groups with more racial diversity^45^. Related effects have emerged in work on ‘identity safety cue transfers’, where people from minority groups use commitments to a specific form of diversity to infer broader egalitarian values. In one example, white female participants’ perceptions of fairness towards women and anticipated inclusion was lower in a company that implemented a general training programme that did not mention race or gender, but these perceptions were ameliorated when the company implemented a training programme that supported either women or racial minorities^46^, with similar effects emerging among male Black and Latino participants. Conversely, female participants anticipated greater sexism from an evaluator displaying racially biased behaviour, and male racial minority participants anticipated greater racial bias from an evaluator engaging in sexist behaviour^47^. This work aligns with separate findings that although some groups are more associated with the concept of diversity than others^48–50^, there can be a sizeable overlap in the shared identity of minorities, especially among members of racial/ethnic minority groups^51–53^. Similarly, individuals with at least one stigmatized identity (such as white women or Black men) may be more sensitive to issues of privilege and respond more positively to all forms of organizational diversity than individuals with no stigmatized identities (that is, white men^12^).

These findings lead to diverging predictions on how the behaviour of minority group members in this field study will be impacted by differing forms of organizational diversity. If willingness to submit an application is primarily tied to seeing an organization hire people sharing one’s own racial/ethnic or gender identity^44^, female white participants should be impacted by seeing organizations with gender but not racial/ethnic diversity, and male non-white participants should be impacted by seeing organizations with racial/ethnic but not gender diversity. However, if the decision to submit an application is sensitive to these identity safety transfer cues, then any form of diversity should impact members from minority groups^46^.

This second perspective would predict that white women, non-white women and non-white men will apply more to organizations that promote any form of racial/ethnic or gender diversity. That is, the analyses should produce a reliable interaction between the racial/ethnic and gender diversity manipulations, such that rates of applying are lower only in the organization made entirely of white men, which lacks both racial/ethnic and gender diversity (Hypotheses 2, 4 and 6 in Supplementary Table 1).

A final question concerns how members of majority groups (here, white men) will be impacted by varying forms of organizational diversity. Prior studies have found that, compared with minority group members, majority group members differ in the cues and information they use to assess organizational diversity^54–56^. Indeed, majority group members have been found to be relatively insensitive to organizational diversity cues^27–29^. For instance, whereas a manipulation that introduced a generic, gender-specific or race-specific training programme impacted beliefs about being treated fairly among female and racial minority participants, white male participants showed no effect across conditions^46^. Similarly, white participants’ intentions to pursue a job were unaffected by messages indicating that the organization had instituted a mentoring programme that was limited to Black employees^57^.

However, separate work has found that members of high-status or majority groups can feel threatened by signs of increased diversity, as white people implicitly and explicitly associate the concept of multiculturalism with that of exclusion (versus inclusion)^58^. In the job-seeking context, past studies have found that white participants applying for a hypothetical job reported more concerns about receiving unfair treatment or less organizational attractiveness when the organization mentioned a commitment to diversity^59,60^, results that align with more general findings of greater threat experienced by white people shown information about increased racial diversity in the US population^61,62^. Moreover, past work has found that white participants considered an organization to be less attractive when the organization’s recruitment website featured a greater proportion of testimonials from minority employees^25^. Finally, correlational research among actual employees has also highlighted lower willingness to embrace organizational diversity among majority group members; one large-scale survey of 2,686 employees revealed that, compared with white women and racial/ethnic minorities, white men reported lower comfort with and perceived value in organizational diversity^38^.

Such reactions towards the concepts of diversity could make it less appealing for members of majority groups to submit applications to organizations that appear to be embracing diversity. From this perspective, one hypothesis is that results would show an adverse reaction among white men to the concept of diversity, finding that white men will apply less to organizations that have racial/ethnic diversity (Hypothesis 7 in Supplementary Table 1) or both racial/ethnic and gender diversity (that is, main effects of both the racial/ethnic and gender diversity manipulations; Hypothesis 8 in Supplementary Table 1).

Compared with applicants with stigmatized identities, it is less clear how members of majority groups (here, white men) will react to manipulations showing varying forms of organizational diversity. There is even the possibility that white men will respond positively to greater organizational diversity, given the prior finding that materials promoting organizational diversity resulted in more positive perceptions from both minority and majority group members^21^. This work then provides an additional test, using a naturalistic and externally valid setting, of how majority group members respond to information related to organizational diversity.

Interestingly, research on diversity and employee recruitment has largely ignored the role of applicant quality^63,64^. However, reactions to diversity cues may vary depending on applicant quality. For instance, previous models suggest that targeted recruitment based solely on demographics may lead to a greater number of applications submitted by less qualified minority jobseekers, thereby weakening the overall quality of applications from minority jobseekers^63^.

The opposite effect may emerge for more qualified applicants with stigmatized identities, as a prior field study found that organizations promoting diversity through an equal employment opportunity statement received fewer applications from more qualified minority applicants. Follow-up surveys suggested that this phenomenon was due to tokenism concerns: highly qualified minority applicants wanted to avoid settings that were more likely to hire for symbolic purposes rather than for merit^65^. To the extent that our own manipulations create similar tokenism concerns, it is possible that they may reduce motivation to apply among more qualified minority applicants. In the present research, it may then be the case that signalling a commitment to diversity by increasing the diversity of the organization’s workforce will increase the number of applications from less qualified stigmatized applicants and decrease the number of applications from more qualified stigmatized applicants. As a result, one hypothesis from prior research is that increased diversity in recruitment materials will result in lower applicant quality from applicants with stigmatized backgrounds who see employees sharing their own racial/ethnic or gender identity. For example, this perspective would anticipate that non-white men will show lower levels of applicant quality when viewing organizations that have any racial/ethnic diversity (that is, a main effect of racial/ethnic diversity; Hypotheses 9–11 in Supplementary Table 1). Accordingly, if white male applicants view diversity as a potential threat that deters less qualified applicants, the average quality of white male applicants may increase when organizations are shown with greater racial/ethnic or gender diversity (Hypothesis 12 in Supplementary Table 1).

This process may produce a somewhat ironic effect of greater diversity in organizational materials leading to weaker overall applicant quality from members of stigmatized groups. We tested this possibility directly by hiring professionals with human resources experience to rate the quality of applicants from both stigmatized and non-stigmatized groups. We recognize that evaluations from individuals with experience in human resources are still subjective, so such analyses cannot speak to any truly objective measure of applicant quality. However, this approach had the dual benefit of both capitalizing on our raters’ prior expertise in evaluating job applicants and closely mirroring the method by which many organizations screen potential employees.

By observing application behaviour in a realistic setting, this work advances understanding of how various signals related to organizational diversity affect the demographic makeup, quality and overall size of an applicant pool. In particular, this study investigated (1) whether prior lab findings that members of minority groups show greater interest in organizations that highlight demographic similarity among current employees^39^ can be reproduced in field settings; (2) whether members of minority groups are impacted by subtle signals of organizational diversity, even when such diversity does not reflect their own identity^46^; (3) whether members of majority groups show a negative reaction (that is, a lower likelihood of submitting an application) to greater organizational diversity^54^; and (4) how the overall level of applicant quality is influenced by organizational diversity cues^65^.

Finally, it is important to consider the appropriate interpretation of possible null effects. If null effects are observed, it would be inaccurate to conclude that organizational diversity has no effect on applicant behaviour or quality. Rather, null effects should only be interpreted as a lack of evidence that this particular manipulation of cues to organizational diversity impacted job applicants. Given the field design of this work, there are various unobservable factors that prevent more specific conclusions regarding null results.

## Results

All analyses employed two-sided tests for statistical significance.

### Sample

Analysis 1, which investigated whether the experimental manipulations impacted the demographics of the applicant pool, included 1,585 applications (281 white men, 183 white women, 686 non-white men and 435 non-white women). Analysis 2, which focused on whether the experimental manipulations impacted applicant quality, used a randomly selected subset of 922 applications (281 white men, 168 white women, 316 non-white men and 157 non-white women). Analysis 3 investigated whether the experimental manipulations impacted the overall likelihood of starting or submitting an application, regardless of participant race/ethnicity or gender. This analysis used all 31,928 unique website visitors (see the Methods for the pre-registered exclusion criteria and power analyses). Participant age was not measured.

### Data quality checks and transformations

The original protocol outlined a concern of potential ceiling effects in the portion of the application that involved a self-assessment of various job-related skill sets, but this did not appear in the actual data, as only 8.26% of participants gave themselves the highest possible rating across all dimensions.

### Organizational diversity and applicant demographics

In Analysis 1, we investigated how our experimental manipulations impacted the demographic makeup of the applicant pool. Specifically, we ran four binary logistic regressions, where we predicted the likelihood of an applicant being a white man, a white woman, a non-white man or a non-white woman from their gender diversity condition (1 = present, 0 = absent), their racial/ethnic diversity condition (1 = present, 0 = absent) and their interaction. Given that applicants accessed the company’s website using the exact same advertisements, we can assume that there were no systematic differences across conditions in terms of the demographic makeup of who was exposed to each condition. As a result, these analyses can identify whether our manipulations of organizational diversity cues made members of certain racial/gender groups more or less likely to apply. None of these four analyses returned a reliable main effect of either racial/ethnic or gender diversity, or an interaction, at the level of *P* < 0.05. See Table 1 for descriptive and sample size statistics for all primary analyses, and see Table 2 for statistical reporting of Analysis 1.

**Table 1:** Descriptive and frequency statistics for Analyses 1–3

Analysis 1: frequency of each subgroup by condition				
Condition	White men	Non-white men	White women	Non-white women
No diversity (*N* = 441)	18.14%	43.76%	11.79%	26.30%
Racial/ethnic diversity (*N* = 338)	19.82%	40.83%	14.20%	25.15%
Gender diversity (*N* = 332)	17.47%	43.37%	11.14%	28.01%
Racial/ethnic and gender diversity (*N* = 474)	16.03%	44.51%	9.70%	29.75%

Analysis 2: average qualification score (s.d.) and sample size for each subgroup and condition				
Condition	White men	Non-white men	White women	Non-white women
No diversity	3.13 (0.93), *N* = 80	3.28 (0.86), *N* = 80	3.70 (0.89), *N* = 44	3.36 (0.81), *N* = 43
Racial/ethnic diversity	3.15 (0.76), *N* = 67	3.15 (0.83), *N* = 77	3.55 (0.67), *N* = 43	3.92 (0.76), *N* = 38
Gender diversity	3.24 (0.84), *N* = 58	3.18 (0.81), *N* = 74	3.47 (0.85), *N* = 37	3.28 (0.61), *N* = 39
Racial/ethnic and gender diversity	3.06 (0.84), *N* = 76	3.15 (0.82), *N* = 85	3.48 (0.84), *N* = 44	3.47 (0.69), *N* = 37

Analysis 3: frequency of applicant behaviours for each condition			
Condition	Job ad opened	Application started	Application submitted
No diversity (*N* = 7,384)	23.67%	16.82%	6.54%
Racial/ethnic diversity (*N* = 8,771)	15.57%	11.10%	4.16%
Gender diversity (*N* = 7,961)	16.46%	12.10%	4.51%
Racial/ethnic and gender diversity (*N* = 7,812)	22.22%	16.40%	6.54%

**Table 2:** Results for Analysis 1: the impact of experimental manipulation on the demographics of the applicant pool

Term	*B* (s.e.)	*P*	OR (95% CI)
Outcome: applicant is a white man			
Racial/ethnic diversity	0.11 (0.18)	0.552	1.12 (0.78, 1.60)
Gender diversity	−0.05 (0.19)	0.810	0.96 (0.66, 1.39)
Racial/ethnic × gender diversity	−0.21 (0.27)	0.423	0.81 (0.48, 1.36)
Outcome: applicant is a non-white man			
Racial/ethnic diversity	−0.12 (0.15)	0.411	0.89 (0.67, 1.18)
Gender diversity	−0.02 (0.15)	0.914	0.98 (0.74, 1.31)
Racial/ethnic × gender diversity	0.17 (0.21)	0.418	1.18 (0.79, 1.77)
Outcome: applicant is a white woman			
Racial/ethnic diversity	0.21 (0.22)	0.320	1.24 (0.81, 1.89)
Gender diversity	−0.06 (0.23)	0.780	0.94 (0.60, 1.47)
Racial/ethnic × gender diversity	−0.37 (0.32)	0.246	0.69 (0.37, 1.29)
Outcome: applicant is a non-white woman			
Racial/ethnic diversity	−0.06 (0.17)	0.715	0.94 (0.68, 1.30)
Gender diversity	0.09 (0.16)	0.597	1.09 (0.79, 1.50)
Racial/ethnic × gender diversity	0.15 (0.23)	0.527	1.16 (0.74, 1.81)

Series of binary logistic regressions predicting applicant demographics from the racial/ethnic diversity condition, the gender diversity condition and their interaction. *B* represents the condition-associated change in the log odds that an applicant is from the specified demographic group. CI, confidence interval.

### Organizational diversity and applicant quality

In Analysis 2, we investigated the effects of our manipulation on applicant quality. Organizational diversity cues could impact whether qualified applicants from majority and minority groups decide to apply. For instance, if individuals from minority groups anticipate unfair treatment in organizations that lack diversity, this may lead to only the most qualified members submitting applications^63^. A similar effect could emerge for majority group members (that is, white men); if they view diversity as a threat, only the most qualified applicants will apply to positions in more diverse organizations.

**Fig. 2:**
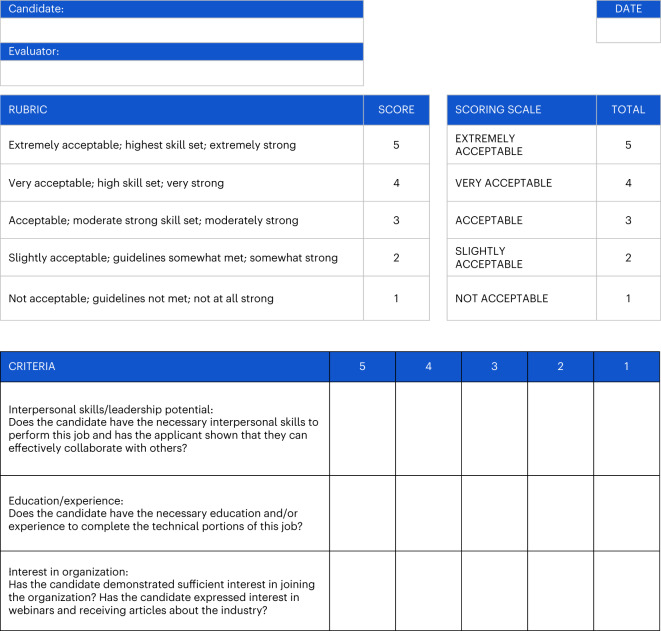
Rubric used by the human resource coders to evaluate applicants. For the original registered rubric, see Supplementary Fig. 20.

Consultation with human resources professionals led to a rubric with three outcome scores, each rated on a 1–5 scale by coders (interpersonal skills/leadership, education/experience and interest in organization), with the overall qualification rating being the average of the three scores (see Fig. 2 and Methods for more information). We ran a series of two (racial/ethnic diversity: absent versus present) by two (gender diversity: absent versus present) between-participant analyses of variance (ANOVAs), with overall applicant score as the dependent variable, separately for white women, white men, non-white women and non-white men. For white men, non-white men and white women, the analyses did not find any reliable main effects or interactions concerning our experimental manipulations at *P* < 0.05. However, for non-white women, the analyses produced main effects for both the gender diversity and racial/ethnic diversity conditions, such that on average applicants were more qualified when viewing an organization with racial/ethnic diversity (mean = 3.70, s.d. = 0.76) than when viewing one without racial/ethnic diversity (mean = 3.32, s.d. = 0.72), and applicants were also more qualified when viewing an organization without gender diversity (mean = 3.62, s.d. = 0.83) than when viewing one with gender diversity (mean = 3.37, s.d. = 0.66). See Table 3 for reporting.

**Table 3:** Results for Analysis 2: the impact of experimental manipulation on applicant quality

Term	*F*	*P*	*η*_p_^2^ (95% CI)
White men applicants (*N* = 281)			
Racial/ethnic diversity	0.58	0.446	0.002 (0, 0.026)
Gender diversity	0.02	0.889	<0.001 (0, 0.003)
Racial/ethnic × gender diversity	1.00	0.317	0.004 (0, 0.031)
Non-white men applicants (*N* = 316)			
Racial/ethnic diversity	0.72	0.398	0.002 (0, 0.019)
Gender diversity	0.35	0.557	0.001 (0, 0.015)
Racial/ethnic × gender diversity	0.27	0.604	0.001 (0, 0.014)
White women applicants (*N* = 168)			
Racial/ethnic diversity	0.30	0.586	0.002 (0, 0.027)
Gender diversity	1.43	0.234	0.009 (0, 0.046)
Racial/ethnic × gender diversity	0.46	0.500	0.003 (0, 0.031)
Non-white women applicants (*N* = 157)			
Racial/ethnic diversity	10.47	0.001	0.064 (0.015, 0.134)
Gender diversity	5.16	0.025	0.033 (0.002, 0.090)
Racial/ethnic × gender diversity	2.65	0.105	0.017 (0, 0.065)

Series of two (racial/ethnic diversity) by two (gender diversity) ANOVAs for each demographic group.

### Organizational diversity and applications started or submitted

A final question concerned whether any of the experimental manipulations resulted in more started or submitted applications. If greater racial/ethnic and gender diversity is equally appealing to both majority and minority group applicants^21^, there may be no impact on the percentage of applicants that come from minority versus majority groups or on the average quality of applicants. However, such an effect would be evident on the overall likelihood of participants submitting or starting an application.

**Fig. 3:**
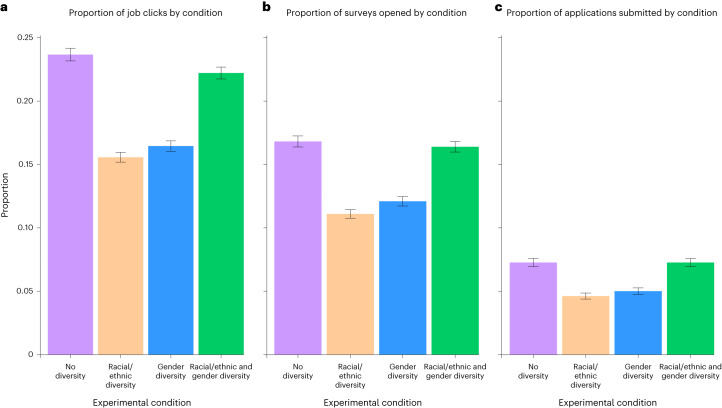
Proportion of job clicks by condition, proportion of surveys opened by condition and proportion of applications submitted by condition. **a**–**c**, Results of Analysis 3, which investigated how the experimental manipulations impacted the likelihood of applicants opening a job ad (**a**), starting an application (**b**) and submitting an application (**c**). The error bars indicate plus or minus one standard error. Source data

This question was the focus of Analysis 3, where we ran a series of binary logistic regressions, predicting for each website visitor whether or not they submitted an application (1 = yes, 0 = no) from the gender diversity condition (1 = absent, 0 = present), the racial/ethnic diversity condition (1 = absent, 0 = present) and their interaction. This analysis found reliable main effects of racial/ethnic diversity and gender diversity, but these were qualified by a significant racial/ethnic-diversity-by-gender-diversity interaction. Specifically, a crossover interaction occurred such that applications were more likely to be submitted in conditions with both racial/ethnic and gender diversity (6.54% of visitors) or neither racial/ethnic nor gender diversity (6.54% of visitors) than in conditions with only gender diversity (4.51% of visitors) or only racial/ethnic diversity (4.16% of visitors). The same pattern emerged when looking only at whether each visitor began an application or opened a job ad. See Table 4 for the full reporting and Fig. 3 for a graphical presentation of the results.

**Table 4:** Results for Analysis 3: the impact of experimental manipulation on application-related behaviour

*Term*	*B* (s.e.)	*P*	OR (95% CI)
Outcome: submitting an application			
Racial/ethnic diversity	−0.48 (0.07)	<0.001	0.62 (0.54, 0.71)
Gender diversity	−0.39 (0.07)	<0.001	0.68 (0.59, 0.78)
Racial/ethnic × gender diversity	0.87 (0.10)	<0.001	2.39 (1.96, 2.91)
Outcome: starting an application			
Racial/ethnic diversity	−0.48 (0.05)	<0.001	0.62 (0.56, 0.68)
Gender diversity	−0.39 (0.05)	<0.001	0.68 (0.62, 0.75)
Racial/ethnic × gender diversity	0.84 (0.07)	<0.001	2.31 (2.03, 2.62)
Outcome: clicking on the job advertisement			
Racial/ethnic diversity	−0.52 (0.04)	<0.001	0.60 (0.55, 0.64)
Gender diversity	−0.45 (0.04)	<0.001	0.64 (0.59, 0.69)
Racial/ethnic × gender diversity	0.89 (0.06)	<0.001	2.44 (2.18, 2.73)

Series of binary logistic regressions predicting various application-related behaviours from the racial/ethnic diversity condition, the gender diversity condition and their interaction.

Given our experimental design, we cannot investigate whether any such effects are moderated by the participant’s own race/ethnicity or gender. This is because such information is reported only during the application process, meaning we lack demographic information from those who decided not to apply for a position. However, as mentioned earlier, all participants saw the same advertisement text when first deciding to visit the company’s website, so there should be no systematic bias in the number of participants from minority or majority groups who viewed each condition.

### Exploratory analysis of gender and applicant quality

Our primary analyses did not focus on demographic differences in applicant quality, regardless of experimental manipulation. However, the descriptive statistics in Analysis 2 suggested gender differences in applicant quality, such that women were rated as more qualified than men. Indeed, an exploratory analysis (collapsing across experimental conditions) using a two (applicant gender: male or female) by two (applicant race/ethnicity: white versus non-white) ANOVA on overall applicant quality found no reliable main effect of applicant race/ethnicity and no interaction between applicant gender and applicant race/ethnicity, but a robust main effect of applicant gender, such that women (mean = 3.53, s.d. = 0.79) were rated as more qualified than men (mean = 3.16, s.d. = 0.84). See Table 5 for the full reporting. This main effect persisted when accounting for experimental manipulation (Supplementary Table 7).

**Table 5:** Results for Exploratory Analysis 4: demographic differences in applicant quality

Overall applicant quality (*N* = 922)			
Term	*F*	*P*	*η*_p_^2^ (95% CI)
Participant race/ethnicity	0.001	0.969	<0.001 (0, 0.0002)
Participant gender	41.81	<0.001	0.044 (0.024, 0.066)
Participant race/ethnicity × gender	0.86	0.355	0.001 (0, 0.007)

Two (participant race/ethnicity: white = 1) by two (participant gender: male = 1) ANOVAs across all coded applications, collapsing across experimental conditions.

### Robustness checks and interpreting null results

As outlined in our Stage 1 protocol, we reviewed the extent to which our conclusions do or do not change when using alternative *α* levels (0.01, 0.005 and 0.001). Only one of our reported analyses had different conclusions when using more stringent *α* levels. The main effect of gender diversity on applicant quality among non-white women in Analysis 2 is no longer significant when using *P*-value cut-offs below 0.01, and the main effect of racial/ethnic diversity in that same analysis is no longer significant when using a *P*-value cut-off below 0.001. All other significant analyses are retained when using a *P*-value cut-off of 0.001.

Finally, we investigated how the confidence intervals surrounding our obtained effect sizes existed relative to the effect sizes targeted in our initial protocol. These discussions are limited to analyses that failed to reject the null hypothesis, and are meant to allow for more meaningful interpretations of any null results. For Analysis 1, the smallest effect size of interest concerned main effects of racial/ethnic or gender diversity on demographic representation in the applicant pool at a level of Cohen’s *d* = ±0.30 (odds ratio (OR), 0.58; OR = 1.72). The 95% confidence intervals on effect sizes in Analysis 1 fell within these bounds for seven of our eight main effects, with the one exception being the main effect of racial/ethnic diversity on the likelihood of an applicant being a white woman. For Analysis 2, the smallest effect size of interest was a main effect of racial/ethnic or gender diversity on applicant quality at a level of Cohen’s *d* = 0.42 (*η*_p_^2^ = 0.042). Of the six main effects that failed to reject the null hypothesis (among white men, white women and non-white men), five had a 95% confidence interval on the effect size falling within this range; the one exception was the effect of gender diversity on applicant quality among white women. Analysis 3 is not included because all results were statistically significant. In general, these results suggest that most of our null results are consistent with a lack of an effect at our pre-specified effect sizes of interest.

## Discussion

The present work used a large sample of actual jobseekers to provide an externally valid test examining how members of minority and majority groups react to cues of organizational diversity. Specifically, this study explored how the presence or absence of racial/ethnic or gender diversity among employees (presented visually on a company website) impacted the quantity and quality of applications. An additional strength of this work is the Registered Report format, as the methods, samples and analyses were pre-registered and approved prior to data collection. This work also uses open science practices, as the study materials, data and code are publicly available online. Our analyses, which often produced unanticipated patterns of results, carry important practical and theoretical implications for research in intergroup relations, organizational behaviour and digital marketing.

Analysis 1 examined whether organizational diversity would influence the number of submitted applications from applicants with different racial/ethnic or gender identities. Given past work on organizational diversity cues, we expected that applicants would be more attracted by (and more likely to apply to) organizations whose employees shared the applicant’s racial/ethnic identity, gender identity or both. However, there was little evidence that the manipulation of organizational diversity—conveyed through images of employees pictured on a company’s website—increased the relative representation of any subgroup in the applicant pool. These findings contrast with prior lab studies using members of stigmatized groups, which have often shown that diversity cues can increase organizational appeal when employee and applicant demographics match^19,28,42^, particularly when employees and applicants are aligned on racial identity^39–41^.

Analysis 2 investigated whether our diversity manipulation affected applicant quality (for example, whether more qualified non-white men would avoid organizations with racial/ethnic diversity for fear of token treatment^65^). The results from Analysis 2 showed little evidence that our manipulation impacted applicant quality, with one exception: non-white women were rated as more qualified when applying to organizations with racial/ethnic diversity but were rated as less qualified when applying to organizations without gender diversity. Notably, this latter effect was not significant when using more conservative levels for rejecting the null hypothesis and should be treated with caution. The main effect of racial/ethnic diversity leading to greater applicant quality for non-white women was more robust but did not emerge for other demographic groups and countered results from prior work^65^. While Analysis 2 failed to produce consistent effects of racial/ethnic or gender diversity on applicant quality, we believe that the unusual and unexpected effect of diversity cues on the quality of non-white women applicants should be further investigated.

A natural question is why our manipulation did not lead to observable changes in applicant quality or the degree to which the applicant pool comprised minority or stigmatized applicants. One explanation concerns the potentially limited strength of our manipulation: since the intervention collapsed across multiple races or ethnicities into a general category of ‘non-white’, it is possible that participants with certain racial/ethnic/gender identities may not have seen themselves as well-represented in the organization. For example, a Black woman participant in our racial/ethnic and gender diversity condition would still have seen only 2 of 16 employees that were also Black women. Stronger manipulations of organizational diversity may be needed to impact the demographics of an applicant pool in naturalistic contexts. Another explanation for the pattern of our results focuses on the field setting. Prior studies that have observed the impact of employee demographics on organizational attractiveness involved hypothetical organizations^39–41^. It is possible that participants’ motivation to find a job overrode any effects of our manipulation. That is, perceptions of the organization may have genuinely changed as a result of the diversity manipulations, but these changes were not strong enough to translate into an unwillingness to forego potential employment.

We believe that both explanations—an inadequate manipulation, or a manipulation that impacts perceptions of an organization but not behaviour—are inconsistent with the results of Analysis 3, which found robust effects on the overall likelihood of opening the job ad link on the company website as well as starting or submitting an application. Whereas Analysis 1 only investigated the proportion of the applications in each condition that came from white versus non-white men and women, Analysis 3 focused on the total number of applications submitted in each condition. As a result, Analysis 1 is unable to identify whether our manipulation had consistent effects on behaviour, regardless of applicant demographics. Indeed, this seems to be the case; since there is no reason to believe that there were systematic differences in whether applicants from various racial/ethnic and gender identities were assigned to each condition, Analysis 3 suggests that the effects of our manipulation were present across the full sample.

In particular, Analysis 3 found an intriguing interaction effect between racial/ethnic and gender diversity on applicant behaviour. Applications were higher in conditions with either no diversity (that is, all white men) or both racial/ethnic and gender diversity than in conditions with only racial/ethnic or only gender diversity. When ignoring the no diversity condition, these results are most consistent with prior studies of spillover effects in diversity judgements^46^, where perceptions of diversity on one dimension can be heightened by diversity on another dimension (for example, greater racial diversity in an organization can lead to perceptions of greater gender diversity^45^). Maximizing diversity by presenting employees of various races/ethnicities and genders may have mutually reinforced perceptions of racial/ethnic and gender diversity themselves, and perhaps even led to heightened perceptions of other forms of diversity, such as in age. To the extent that diversity would be appealing to members of stigmatized or minority groups^39–41^, it is then plausible that these spillover effects would have increased applications from white women, non-white men and non-white women for the condition showing racial/ethnic and gender diversity.

At the same time, Analysis 3 suggests that a similar pattern also occurred for white male applicants. Again, when ignoring the no diversity condition, white men were more likely to apply to organizations with more than less diversity. Prior experimental and correlational data have found that white men were either unaffected or threatened by organizations with more racial or gender diversity^25,46,57–62,66^, though one recent series of studies produced a conflicting pattern; specifically, presenting white American participants with organizations advocating for multiculturalism over colour blindness increased perceptions of the organization’s commitment to diversity but had no impact on feelings of social identity threat or feelings of inclusion^67^. These latter results also align with national survey data showing that white Americans have become increasingly supportive of diversity efforts in the past decade^67^. If similar processes occurred in the present data, white men could also have been more drawn to the organization that showed both racial/ethnic and gender diversity. In all, though a majority of the prior literature would anticipate that white men would react negatively to suggestions of a commitment to diversity, recent analyses provide more mixed evidence, and the present work suggests conditions where greater racial/ethnic and gender diversity could be more appealing to white male applicants.

However, these discussions ignore the most unexpected result from Analysis 3, where an organization with no racial/ethnic or gender diversity led to more applications than organizations with either racial/ethnic or gender diversity, an effect that emerged across applicant demographics. We lack a definitive explanation for this effect and do not believe that it has a clear precedent in the literature. One potential explanation is that the no diversity condition could have been viewed as merely reflecting the status quo of privileging white men in the tech industry^68–70^. As a result, participants may have believed that the company was engaging in ‘passive discrimination’ by simply relying on industry-wide defaults in hiring practices. These reactions could have diverged from perceptions of the organizations presented in the racial/ethnic or gender diversity conditions, who could have been seen as engaging in more ‘active discrimination’ by hiring employees from one minority group but not another. This active discrimination may have led to lower levels of organizational attractiveness, resulting in fewer applications. Similarly, the all-white, all-male and fully diverse organizations may have been viewed as more authentic or realistic, while the organizations presenting only one form of authenticity may have deviated from participants’ expectations and resulted in lower appeal. We concede that these are speculative accounts of our results, and more data are needed to verify this explanation; for instance, follow-up work could explore whether the organization with no diversity is less appealing to potential applicants only when they are provided with evidence that the company has been accused of discrimination in their hiring practices.

A final, exploratory analysis emerged outside of our intervention. Female applicants were rated as more qualified than male applicants, even after controlling for experimental condition. This finding may have emerged as a result of multiple processes. For one, more qualified male applicants could already be employed, as biases favouring male applicants in a male-dominated field^71^ would have left more qualified female jobseekers in our applicant pool. In addition, this result may have been driven by more internal factors. Prior research has found that men score higher in overconfidence^72^ and competitiveness^73^ than women, which could have led a greater number of moderately qualified or unqualified men to apply. Though the precise mechanism behind this effect is unclear and its exploratory nature requires confirmatory evidence, the robust effect should be of interest to researchers studying discrimination as well as practitioners looking to increase fairness in hiring.

While the field context of our study has several advantages, the study does carry several limitations. First, the distribution of applicants from various racial/gender identities deviated from what was anticipated in the Stage 1 protocol. In particular, the final applicant pool had fewer applications from white men and white women than was expected, meaning that our achieved statistical power for each racial/ethnic–gender group in Analysis 1 diverged from our original estimates. At the same time, we exceeded our target number of total applications, so while our statistical power for white men and white women was lower than planned, power was higher than planned for non-white men and non-white women.

Another limitation is the simplification of racial or ethnic identities into a white versus non-white categorization. This decision was necessary to achieve sufficient statistical power and is consistent with prior work showing evidence of broader solidarity among members of stigmatized groups^51,53^, but it precludes more fine-grained analyses that focus on a particular non-white racial identity (for example, Black men or Black women). Subsequent work could provide a more detailed analysis across identities that have been underexplored here due to reasons of statistical power. For instance, such work could explore how organizational diversity cues affect jobseekers that are Asian, Black or Hispanic. Such an analysis would help differentiate the effects of diversity cues on having both a stigmatized and underrepresented identity, as Asian people are well represented in the technology industry compared with the general population^70^. Our open dataset may be a useful resource in conducting initial analyses on this question. Relatedly, future work could explore how different forms of organizational diversity—such as those based on religion, political affiliation or sexual orientation—impact applicant behaviour.

Moreover, our field approach did not allow us to collect individual difference measures, such as openness to diversity or centrality of gender identity, which have emerged in prior work as helpful for identifying who will have more positive or negative reactions to organizational diversity^26,30^. Including such measures may have better clarified our results, but adding them to a field study could have aroused participant suspicion and compromised our goal of observing naturalistic behaviour. A final limitation is that all job postings were listed as remote to adjust to the context of the ongoing COVID-19 pandemic. Remote positions may have led to unique considerations when evaluating organizations, and future work should investigate whether similar results emerge for in-person job postings. However, our results are at a minimum still highly relevant to the many organizations that are shifting to greater levels of remote employment.

Our large-scale field test of the influence of employee demographics on applicant behaviour provides externally valid data that extend existing work in domains such as organizational attraction or attachment, intergroup threat, digital marketing and perceptions of diversity. These results also have practical implications for organizations looking to promote the recruitment of applicants from minority groups or to effectively signal their commitment to diversity. For one, our work counters prior explanations that failings in recruitment of applicants from minority or stigmatized groups stems from a ‘pipeline problem’, as we observed large numbers of applications from non-white and female jobseekers, with female applicants even being rated as more qualified than their male counterparts. Second, we found that people from different backgrounds tended to have similar reactions to our organizational diversity manipulation. Finally, and most notably, our results showed little evidence that merely presenting more diverse workforces increased applications from stigmatized or minority group members. Organizations may thus be well served to search for alternative methods that eschew such surface-level approaches, instead adopting practices that signal stronger and more meaningful commitments to creating a diverse workforce. Taken together, our work suggests that digitally displaying a diverse workforce is not sufficient to foster consistent improvements in the recruitment of stigmatized groups.

## Methods

R software (v 4.2.2) was used to clean and de-identify the dataset and to complete the a-priori power calculations while SPSS (v 27) was used to conduct our analyses.

### Ethics

This study received approval from the Duke University Institutional Review Board (protocol no. 2018-0264). Given the field setting, we obtained waivers for consent, compensation and debriefing. Identifiable data are stored on password-protected computers, while de-identified data and analysis code are accessible at https://osf.io/vaq2g/. See Supplementary Table 2 for the details and links for each resource available in the online repository.

### Data collection

Data collection began on 25 February 2021 and was paused between 7 May 2021 and 23 June 2021 to accommodate an additional ethics review of our protocol in the context of the COVID-19 pandemic and to amend our protocol to allow for data collection through an additional job recruitment platform (Indeed). While we were requesting a protocol amendment to expand recruitment to the additional platform, a reviewer queried whether the COVID-19 pandemic may have shifted the balance between the benefits and harms of our study, which involved soliciting applications for jobs at a fictional firm. We therefore sought further review from our Institutional Review Board, which reaffirmed its approval of the study. In light of this second approval and the shifting impacts of the pandemic over time, we attempted to expand recruitment to Indeed.com on 14 July 2021 and later resumed data collection. Ultimately, however, the job postings on Indeed did not pass quality controls, and we were unable to recruit participants from that source.

### Deviations from Stage 1 protocol

We report four deviations from our Stage 1 protocol. First, for Analysis 1 and Analysis 2, we excluded participants who spent less than 15 seconds on the company webpage (6.90% of applications), whereas the original cut-off was less than 30 seconds (40.62% of applications). We made this deviation after considering the very high exclusion rate for the 30-second criterion and deciding that 15 seconds was still a sufficient amount of time spent on the page. Supplementary Tables 3 and 4 report the results of Analyses 1 and 2 using the 30-second criterion. Second, we simplified the criteria used to evaluate applicant quality from four to three domains on the basis of feedback from the raters (see ‘Applicant evaluation’ for more details). Third, to conduct Analysis 2, we evaluated a greater number of applications from non-white men and non-white women, and we evaluated a smaller number of applications from white men than we initially pre-registered. Contrary to the assumptions of our initial submission, our applicant pool was mostly non-white (~71%). As a result, we met or exceeded the original target number of graded applications for white women, non-white women and non-white men. We originally anticipated grading 300 white men applicants but fell short of this target despite grading all white men applicants (281). Finally, we were unable to complete formal equivalence tests given that existing software does not support such tests for the multigroup designs used here. Instead, we discuss how our targeted effect sizes (that is, the smallest effect size of interest specified in our Stage 1 submission) existed relative to the confidence intervals on effect sizes produced from our actual analyses (see ‘Robustness checks and interpreting null results’ for more details).

### Procedure

#### Participant recruitment

We recruited participants online by posting up to nine jobs on LinkedIn and promoting them with a daily budget between US$15 and US$35. The nine jobs were at a company called Foodable, which was a “Silicon Valley based company on a mission to redefine how people purchase and consume food”. The job posts prompted potential applicants to apply directly on the company website, which presented basic background information about the company, displayed an image showing current employees and listed job openings. See Supplementary Figs. 1–19 for the website content and job descriptions.

#### Experimental manipulation

After clicking the advertisement, the participants were randomly assigned to one of four experimental conditions, following a two (racial/ethnic diversity: yes versus no) by two (gender diversity: yes versus no) design. The experimental manipulation of organizational diversity was conveyed through an image shown on the ‘Team’ heading of the website, as well as through an image on the bottom of the page. The participants were randomized to experimental condition after opening the website link.

To minimize the risk of exposure to multiple website conditions, condition assignment was maintained utilizing the participant’s HTTP cookies and their IP address. In the background of their browsers, the participants were sent HTTP cookies by accessing the website. Critically, the website linked a participant’s HTTP cookies and IP address to their condition. This two-factor authentication ensured that every new visitor that had the same HTTP cookies and/or the same IP address would see the version of the website they saw at the first visit. If the participant disabled HTTP cookies or cleared their cookies between visits, they viewed the website condition assigned to their IP address. Ultimately, maintaining assignment using IP addresses helped prevent participants from detecting the condition within networks (that is, across devices at home, at the office or at the library), while also maintaining assignment with HTTP cookies helped prevent participants from detecting the condition between networks when the same device–browser combination was used (for example, accessing the website from Google Chrome on a mobile phone at home and then later at work). For more details about the condition assignment procedures, see the link to ‘Condition.Assignment.Maintenance’ in Supplementary Table 2.

Each condition showed 16 team members presented with headshots and names. See Fig. 1 for the stimuli from each condition. In the no racial/ethnic diversity, no gender diversity condition, the team consisted entirely of white males. In the no racial/ethnic diversity, gender diversity condition, the team consisted entirely of white people but had eight men and eight women. In the racial/ethnic diversity, no gender diversity condition, the team consisted entirely of men, with eight white men and eight men from racial/ethnic minority backgrounds (three Black, two East Asian, two South Asian and one Hispanic). Finally, in the racial/ethnic diversity, gender Diversity condition, the team had four members for each combination of man versus woman and racial/ethnic minority versus white. Racial/ethnic minority team members were given common surnames commonly associated with that racial/ethnic group (for example, Garcia, Nguyen and Gupta). Similarly, team members were given first names that are typically associated with men or women (for example, Michael, Jake and Jason versus Danielle, Dawn and Josephine).

#### Application submission

Across conditions, the website listed up to nine job openings: senior product manager, marketing communications manager, embedded systems architect, account manager, web developer, copy writer, business analyst, recruiter and full stack engineer. These positions were chosen due to their relevance within a technology company. In an effort to increase the sample size, the positions included a range of possible skill sets and were posted online as needed. This paper reports analyses collapsed across positions, while all position-level data are available online. Notably, all potential applicants needed to scroll past the ‘Team’ section of the website before being able to access information about job openings. After clicking on each job title, in-depth information about each position was displayed (responsibilities, qualifications and benefits). We consulted with people who had experience working in each field to write the job descriptions (Supplementary Figs. 5–13).

When viewing each position, participants selected the ‘Apply Now’ button to begin the application process. The application was completed entirely online and did not require the participants to upload a resume. The participants were informed that they may be asked to provide a resume at a later time. See Supplementary Figs. 14–19 for a sample application. After first providing basic contact information, the participants reported their educational background, educational performance (that is, GPA), and performance in math and science classes. The participants then reported any awards or honours they had received, a self-assessment of their performance as a student and their total years of work experience. The participants then reported their work background, information about their previous job titles and responsibilities, length of prior employment, and a self-assessment of effectiveness for their previous jobs and internships. Next, the participants provided ratings of their proficiency in various job-related skills (for example, JavaScript programming), indicated their willingness to review some background information about the company and listed their available times for a possible follow-up interview. Finally, the participants indicated how they had heard about the job advertisement (that is, through an online advertisement, an online search or a referral) before reporting their gender and race and submitting their application. For analytic purposes, we collapsed across anyone who reported race as not white into the ‘non-white’ race/ethnicity category, and we excluded participants who reported gender as ‘Other’, though all participants are available in the online datasets.

#### Applicant evaluation

To develop a system of evaluating applicants, we worked with professionals who had experience in human resources to create an evaluation rubric (see Fig. 2 for the final rubric and Supplementary Fig. 20 for the pre-registered rubric). The rubric was designed to provide an overall assessment of each applicant that could be completed in less than 10 minutes.

After data collection finished, we hired two new coders that also had experience in human resources. The coders were trained together and asked to review groups of sample applications from a pilot study until they achieved acceptable reliability (*I* > 0.60). Specifically, we collected applications from 30 MBA students to train our coders. The two coders assessed application quality for 20 applications randomly selected from our training sample. Inter-rater reliability (*i*) was less than 0.60; therefore, we had the coders meet to review their 20 assessments and discuss the alignment of grading strategies. Afterwards, the coders reviewed the remaining 10 applications, and inter-rater reliability was assessed for these 10 applications (*i* = 0.68).

Once the evaluators achieved acceptable levels of inter-rater reliability, each of the actual applicants was assigned to one of the two coders. The coders were blind to experimental condition and were operating under the assumption that some of these applicants may actually be hired for a position. Using a 1 (not acceptable) to 5 (highly acceptable) scale, the coders rated each applicant on the following dimensions: interpersonal skills, education/knowledge, knowledge and skills in research, and leadership and collegiality.

While attempting to align the grading strategies of our raters, the two human resources experts revealed ambiguities in our grading rubric. For example, ‘knowledge’ was initially a component of two separate dimensions. To increase inter-rater reliability and reduce noise in our quality scale, we deviated from the original protocol and implemented a rating rubric with three dimensions: education/experience, interpersonal skills/leadership potential and interest in the organization. Each dimension was rated using a 1 (not acceptable) to 5 (highly acceptable) scale, and the three dimension scores were averaged to create a total qualification score.

Importantly, the coders evaluated de-identified applications (that is, removing names and email addresses) that were also blinded for race/ethnicity and gender. Application de-identification helped protect the participants’ personal information, while application blinding helped ensure that any biases specific to the raters are spread equally across applicants, regardless of race/ethnicity or gender. However, this blinding of applicant demographic information could have been compromised if the applicant attended a historically Black college or university (HBCU) or an all-women’s college. To address this issue, an author first screened all eligible applications for whether the applicant attended an HBCU or an all-women’s college. Deviating from our original protocol, we expanded screening to identify any federally recognized minority serving institution. In total, 29 applicants attended an HBCU, a minority serving institution or an all-women’s college. For these applicants, their school was replaced with a school of equal or comparable ranking according to 2022 college rankings from the US World and News Report.

### Participants

Our sample size calculations were complicated by the anticipated unequal distribution in applications received from white versus non-white employees. As LinkedIn did not publish information regarding the gender or racial/ethnic breakdown of their users, we relied on estimates from a 2016 report on the demographic composition of tech industry workers (Supplementary Table 2), which indicated the following distributions: 41.3% white men, 16.2% white women, 28.5% non-white men and 14.0% non-white women^68^. Using this distribution as an estimate for our own sample size, we ran a series of power analysis simulations separately for white men, non-white men, white women and non-white women for our focal analysis concerning a logistic regression on the likelihood of being a member of the applicant pool based on organizational gender and racial/ethnic diversity (Supplementary Table 2).

Given our competing hypotheses of whether to expect main effects of either racial/ethnic or gender diversity or interactions between the two factors, we provided power calculations for both outcomes. For non-white men, a sample size of 720 total eligible applicants (that is, from both white and non-white men and women) would provide 95.6% power to detect a small-to-medium main effect (OR = 1.72, Cohen’s *d* = 0.30) of racial/ethnic diversity^74^. For non-white women, a sample of 1,036 participants would provide 95.9% power to detect a main effect of racial/ethnic diversity of the same magnitude. For white women, a sample size of 908 would provide 97.5% power to detect a gender diversity main effect of OR = 1.72. Finally, for white men, a sample size of 944 would achieve 96.1% power for detecting a main effect of racial/ethnic diversity of OR = 0.58 (Cohen’s *d* = 0.30), since racial/ethnic diversity may suppress applications among white men.

The largest number from the above analyses is *N* = 1,036 for the non-white woman analysis. As a result, we considered 1,036 applicants to be our absolute minimum sample size. This sample would be adequately powered to detect theoretically relevant effects of racial/ethnic and gender diversity on applicant recruitment, and null results would be informative for ruling out possible effects at reasonable effect size estimates (Cohen’s *d* = 0.30).

We considered this effect size to be the lower limit for testing a theoretically and practically meaningful intervention. For one, this intervention aimed at impacting only the initial level of the hiring process (that is, the size and makeup of the applicant pool). Job applicants were exposed to many more levels of evaluation, and final hiring decisions are typically based on in-depth evaluations, such as interviews, of only a handful of applicants. As a result, any intervention concerning the first step of the hiring process (that is, submitting an application) would have to be at least moderate in size to effectively carry over into meaningful changes in later stages of hiring. Second, the intervention would need to have at least a moderate effect size to convince organizations to adopt similar practices; many organizations may not be willing to change their employee recruiting processes if the results of this study produced only negligible changes to the applicant pool. Our power analyses were also sensitive to detect effects of similar magnitude in the research literature. For example, Black female participants reported more anticipated belonging in fictional schools that presented Black versus white faculty profiles (*d* = 0.39)^39^, with the same effect size emerging among Latino participants seeing a profile of a Latino employee (*d* = 0.39)^41^.

However, while we considered 1,036 participants to be an absolute minimum, we sought to collect up to 1,524 participants. This number was based on a series of follow-up simulations concerning a possible interaction effect between our racial/ethnic and gender diversity manipulations (specifically, such that the impact of diversity on the likelihood of submitting an application would be equal across organizations that had any form of diversity). Achieving approximately 95% power for detecting a medium interaction effect (OR = 2.07 or OR = 0.48, Cohen’s *d* = 0.40) ranged between 1,496 and 1,524 depending on the demographic group. See Supplementary Table 2 for syntax to run each sample size calculation. As a result, while 1,036 was our minimum sample size, we attempted to continue collecting data until we reached a sample size of *N* = 1,524.

The *N* = 1,036 target sample size also provided sufficient power for other planned analyses. Our second proposed analysis concerned applicant quality. For this analysis, we hired trained coders to rate the applicants. Using the above sample size estimates, coding the 1,036 expected applicants would be prohibitively expensive. Instead, we planned to have the coders rate a subset of applicants. To maximize statistical power, we originally planned to have the coders rate all female and non-white male applicants, which would have achieved 95% power for detecting a main effect of either racial/ethnic or gender diversity of *d* = 0.60 among non-white women, *d* = 0.57 among white women and *d* = 0.42 among non-white men. We also expected to code a random sample of 75 white men per experimental condition, which would have provided 95% power to detect a main effect of racial/ethnic or gender diversity at *d* = 0.42.

However, the applicant demographics deviated considerably from our expectations, and to maximize statistical power, we adjusted our plans for coding applicant quality. Specifically, the coders rated 281 white men (all white men applicants), 316 non-white men, 168 white women and 157 non-white women. This sample provided 95% power for detecting a main effect of either racial/ethnic or gender diversity of *d* = 0.40 among non-white men, *d* = 0.43 among white men, *d* = 0.56 among white women and *d* = 0.58 among non-white women.

Finally, our third analysis concerned whether the experimental manipulations impacted overall rates of submitting an application. Assuming a modest baseline of 3% of website visitors submitting an application, the targeted sample would have provided over 95% power to detect an OR effect size as small as OR = 1.42 for a main effect of either racial/ethnic or gender diversity or an interaction between the two factors, an effect size that is below the benchmark of a small effect (OR = 1.68) (ref. ^74^).

### Data treatment and analysis

#### Exclusions

Analyses 1 and 2 focused on website visitors who submitted a full application, while Analysis 3 focused on all website visitors. Thirteen participants were excluded from Analyses 1–3 for circumventing the manipulation and bypassing the webpage to directly access the application platform. In Analyses 1 and 2, participants were also excluded for (1) spending less than 15 seconds on the website, (2) submitting clearly false applications (for example, listing over 100 years of work experience), (3) indicating ‘Other’ as their gender or (4) indicating that they had heard about the company through a referral. We excluded 121 participants (6.90% of applications) for spending less than 15 seconds on the website. We originally planned to remove participants that spent less than 30 seconds on the website, but we lowered this to a minimum of 15 seconds (‘Deviations from Stage 1 protocol’). We excluded 22 participants for indicating ‘Other’ as their gender and 2 participants for submitting a clearly fake application. We excluded 26 participants for indicating that they had heard about the job through a referral to control for word-of-mouth exposure, attention and manipulation avoidance. Given the possible overlapping exclusion criteria, 168 total submitted applications were excluded from the sample. It was hard to anticipate how many exclusions there would be, if any. However, when such cases arose, we conducted our primary analyses on the full sample and the sample with excluded participants, and the results from the full sample are reported in the Supplementary Information. Since our exclusions did not substantively change the results, only the analyses on the sample with exclusions are reported in the main text. Importantly, these exclusion criteria were applied within the measurement period (from the participant’s first visit to their subsequent application submission).

In addition to the circumstances outlined above, having multiple visitors with the same IP address could complicate our conclusions. To explore this issue, we also re-ran our analyses excluding cases where a new visit was logged from a duplicate IP address after that IP address had already submitted an application; this process excluded any new applicant that came from an identical IP address. Although it did not occur often (2.84% of eligible applications), it was reasonable for a participant with a variety of skills to apply to multiple positions. We therefore planned to exclude such participants from the final sample only if our results were substantively different after duplicate IP addresses were excluded. The conclusions did not change when excluding such applicants. Supplementary Tables 8–10 report analyses restricted to the first time an IP address visited the site (the primary conclusions do not change).

#### Data quality checks and transformations

We did not anticipate having to make any transformations on our data. However, one potential concern was that participants’ self-ratings of their skills would have ceiling effects due to the desire to create a strong impression. To further examine this issue, we ran a pilot study among MBA students (*N* = 24) and asked them to complete the online application. When evaluating their own skill sets, only 9% (11/122) of responses were the highest possible value, and no single skill had more than 17% of responses being the highest value. We expected similar distributions in our field sample and ran a preliminary analysis investigating what percentage of the sample reported having the highest aggregate level of a certain skill. Since no aggregate skill showed ceiling effects (specifically, where more than 40% of participants reported the highest possible value), we did not remove that variable from the data.

### Reporting summary

Further information on research design is available in the Nature Portfolio Reporting Summary linked to this article.

### Supplementary information

Supplementary Information Supplementary Tables 1–10, Figs. 1–20 and figure credit lines.

Reporting Summary

### Source data

Source Data Fig. 3 Dataset for Analysis 3 parts 1–3 and Fig. 3, including the proportion of job ad clicks by condition (Sheet 1 and Fig. 3a), the proportion of survey opens by condition (Sheet 2 and Fig. 3b) and the proportion of survey submissions by condition (Sheet 3 and Fig. 3c).

## Data Availability

We have shared all key materials on the Open Science Framework at https://osf.io/vaq2g/. Our primary analyses focused on how the experimental condition impacts the quantity, quality and diversity of applicants, and we have shared the data needed to reproduce those analyses. However, due to confidentiality concerns, we did not store certain variables (for example, names and email addresses), nor were we able to make certain variables publicly available (for example, undergraduate institutions and names of prior companies where employed). The data were de-identified for analysis using R software (v. 4.2.2), while the effect sizes were calculated using SPSS (v. 27) software. Source data are provided with this paper.

## Code availability

We have shared the data cleaning and analysis code as well as source code for creating the websites on the Open Science Framework at https://osf.io/vaq2g/. Note, due to confidentiality concerns, certain variables are not publicly available.
