# Supplementary material for: A field study of the impacts of workplace diversity on the recruitment of minority group members
Source: Nat Hum Behav. 2023 Oct 30;7(12):2212–27. doi: 10.1038/s41562-023-01731-5 (PMC10730395; doi:10.1038/s41562-023-01731-5)
Supplement: Supplementary file 1 — Supplementary Tables 1–10, Figs. 1–20 and figure credit lines. [file 41562_2023_1731_MOESM1_ESM.pdf]

nature human behaviour

Registered Report

https://doi.org/10.1038/s41562-023-01731-5

# A field study of the impacts of workplace diversity on the recruitment of minority group members

In the format provided by the authors and unedited

Table of Contents


SUPPLEMENTARY TABLES ............ 2

SUPPLEMENTARY TABLE 1. HYPOTHESES, ANALYSIS PLAN, AND POWER ANALYSES ............ 2
SUPPLEMENTARY TABLE 2. RESOURCES AVAILABLE IN ONLINE REPOSITORY ............ 24
SUPPLEMENTARY TABLE 3. ANALYSIS OF APPLICANT DEMOGRAPHICS, 30-SECOND CUTOFF ............ 27
SUPPLEMENTARY TABLE 4. ANALYSIS OF APPLICANT QUALITY, 30-SECOND CUTOFF ............ 28
SUPPLEMENTARY TABLE 5. ANALYSIS OF APPLICANT DEMOGRAPHICS, NO EXCLUSIONS ............ 29
SUPPLEMENTARY TABLE 6. ANALYSIS OF APPLICANT QUALITY, NO EXCLUSIONS ............ 30
SUPPLEMENTARY TABLE 7. ANALYSIS OF GENDER QUALITY EFFECT ............ 31
SUPPLEMENTARY TABLE 8. ANALYSIS OF APPLICANT DEMOGRAPHICS, EXCLUDING DUPLICATE IP VISITS ............ 32
SUPPLEMENTARY TABLE 9. ANALYSIS OF APPLICANT QUALITY, EXCLUDING DUPLICATE IP VISITS ............ 33
SUPPLEMENTARY TABLE 10. ANALYSIS OF APPLICATIONS STARTED OR SUBMITTED, LIMITING TO ONE ROW PER PARTICIPANT (NO REPEAT ACTIONS) ............ 34

SUPPLEMENTARY FIGURES ............ 35

SUPPLEMENTARY FIGURES. WEBSITE CONTENT ............ 35
SUPPLEMENTARY FIGURE 1. WELCOME SCREEN ............ 35
SUPPLEMENTARY FIGURE 2. 'ABOUT US' SECTION ............ 36
SUPPLEMENTARY FIGURE 3. SAMPLE 'TEAM' SECTION ............ 37
SUPPLEMENTARY FIGURE 4. 'WE'RE HIRING' SECTION ............ 38
SUPPLEMENTARY FIGURES. JOB DESCRIPTIONS ............ 39
SUPPLEMENTARY FIGURE 5. SENIOR PRODUCT MANAGER ............ 39
SUPPLEMENTARY FIGURE 6. MARKETING COMMUNICATIONS MANAGER ............ 40
SUPPLEMENTARY FIGURE 7. EMBEDDED SYSTEMS ARCHITECT ............ 41
SUPPLEMENTARY FIGURE 8. FULL STACK ENGINEER ............ 42
SUPPLEMENTARY FIGURE 9. WEB DEVELOPER ............ 43
SUPPLEMENTARY FIGURE 10. ACCOUNT MANAGER ............ 44
SUPPLEMENTARY FIGURE 11. BUSINESS ANALYST ............ 45
SUPPLEMENTARY FIGURE 12. COPYWRITER ............ 46
SUPPLEMENTARY FIGURE 13. RECRUITER ............ 47
SUPPLEMENTARY FIGURES. APPLICATION PORTAL ............ 48
SUPPLEMENTARY FIGURE 14. APPLICATION PORTAL, PART 1 ............ 48
SUPPLEMENTARY FIGURE 15. APPLICATION PORTAL, PART 2 ............ 49
SUPPLEMENTARY FIGURE 16. APPLICATION PORTAL, PART 3 ............ 50
SUPPLEMENTARY FIGURE 17. APPLICATION PORTAL, PART 4 ............ 51
SUPPLEMENTARY FIGURE 18. APPLICATION PORTAL, PART 5 ............ 52
SUPPLEMENTARY FIGURE 19. APPLICATION PORTAL, PART 6 ............ 53
SUPPLEMENTARY FIGURES. ORIGINAL APPLICANT QUALITY RUBRIC ............ 54

FIGURE CREDIT LINES ............ 55

FIGURE CREDIT LINES ............ 55-56

## SUPPLEMENTARY TABLES

**Supplementary Table 1. Hypotheses, Analysis Plan, and Power Analyses**

| Question | Hypothesis | Sampling plan | Analysis plan* | Interpretation of different outcomes |
|---|---|---|---|---|
| Question 1: How do members of a stigmatized group (White women) respond to recruitment materials displaying racial/ethnic diversity, gender diversity, or both racial/ethnic and gender diversity? | Hypothesis 1: White women will apply more to organizations that are presented as having any gender diversity. | Data collection will continue until a minimum of 1036 eligible applications are submitted, which is expected to provide greater than 95% power to detect an OR = 1.72 (*d* = .30) main effect of gender or racial/ethnic diversity. | Analysis 1: Logistic regression predicting the likelihood of applicant being a White woman from gender diversity condition, racial/ethnic diversity condition, interaction between conditions, and dummy-coded variable controlling for position applied for. See https://osf.io/8g65r/, lines 8-14 and 29-38. | 1) A main effect of gender diversity, such that application rates are higher in conditions with gender diversity, would suggest White female applicants respond positively to seeing ingroup gender diversity reflected in organizational materials.<br><br>2) A main effect of gender diversity and a main effect of racial/ethnic diversity, such that racial/ethnic and gender diversity is associated with higher application rates, would suggest White female applicants respond positively to seeing both ingroup gender and outgroup racial/ethnic diversity reflected in organizational materials.<br><br>3) Null results showing |

| | | | | |
|---|---|---|---|---|
| | | | | neither main effect of gender nor racial/ethnic diversity would suggest no evidence that our manipulations impacted application rates of White women. |
| Question 1: How do members of a stigmatized group (White women) respond to recruitment materials displaying racial/ethnic diversity, gender diversity, or both racial/ethnic and gender diversity? | Hypothesis 2: White women will apply more to organizations that are presented as having any level of either racial/ethnic or gender diversity. | Data collection will continue until a minimum of 1524 eligible applications are submitted, which is expected to provide greater than 95% power to detect an OR = 2.07 ($d$ = .40) interaction between racial/ethnic and gender diversity. | Analysis 1: Logistic regression predicting the likelihood of applicant being a White woman from gender diversity condition, racial/ethnic diversity condition, interaction between conditions, and dummy-coded variable controlling for position applied for. See https://osf.io/8g65r/, lines 8-14 and 29-38. | 1) See above for interpretations of main effects.<br>2) An interaction between gender and racial/ethnic diversity would suggest that, for White females, the impact of the gender diversity manipulation depends on the presence or absence of the racial/ethnic diversity manipulation.<br>3) Null results showing no gender by racial/ethnic interaction would suggest that, for White females, there was no evidence that the presence of the racial/ethnic diversity manipulation moderated the impact of the gender diversity manipulation (and vice versa). |

| | | | | |
|---|---|---|---|---|
| <u>Question 2:</u> How do members of a stigmatized group (non-White men) respond to recruitment materials displaying racial/ethnic diversity, gender diversity, or both racial/ethnic and gender diversity? | <u>Hypothesis 3:</u> Non-White men will apply more to organizations that are presented as having any racial/ethnic diversity. | Data collection will continue until a minimum of 1036 eligible applications are submitted, which is expected to provide greater than 95% power to detect an OR = 1.72 (*d* = .30) main effect of racial/ethnic diversity. | <u>Analysis 2:</u> Logistic regression predicting the likelihood of applicant being a non-White man from gender diversity condition, racial/ethnic diversity condition, interaction between conditions, and dummy-coded variable controlling for position applied for. See https://osf.io/8g65r/, lines 8-14 and 40-49. | 1) A main effect of racial/ethnic diversity, such that application rates are higher in conditions with racial/ethnic diversity, would suggest non-White male applicants respond positively to seeing ingroup racial/ethnic diversity reflected in organizational materials.<br><br>2) A main effect of gender diversity and a main effect of racial/ethnic diversity, such that racial/ethnic and gender diversity is associated with higher application rates, would suggest non-White male applicants respond positively to seeing both ingroup racial/ethnic and outgroup gender diversity reflected in organizational materials.<br><br>3) Null results showing neither main effect of gender nor racial/ethnic diversity would suggest no evidence that our manipulations impacted application rates of non- |

| | | | | |
|---|---|---|---|---|
| | | | | White men. |
| <u>Question 2:</u> How do members of a stigmatized group (non-White men) respond to recruitment materials displaying racial/ethnic diversity, gender diversity, or both racial/ethnic and gender diversity? | <u>Hypothesis 4:</u> Non-White men will apply more to organizations that are presented as having any level of either racial/ethnic or gender diversity. | Data collection will continue until a minimum of 1524 eligible applications are submitted, is expected to provide greater than 95% power to detect an OR = 2.07 (*d* = .40) interaction between racial/ethnic and gender diversity. | <u>Analysis 2:</u> Logistic regression predicting the likelihood of applicant being a non-White man from gender diversity condition, racial/ethnic diversity condition, interaction between conditions, and dummy-coded variable controlling for position applied for. See https://osf.io/8g65r/, lines 8-14 and 40-49. | 1) See above for interpretations of main effects.<br>2) An interaction between gender and racial/ethnic diversity would suggest that, for non-White males, the impact of the racial/ethnic diversity manipulation depends on the presence or absence of the gender diversity manipulation.<br>3) Null results showing no gender by racial/ethnic interaction would suggest that, for non-White males, there was no evidence that the presence of the racial/ethnic diversity manipulation moderated the impact of the gender diversity manipulation (and vice versa). |

| | | | | |
|---|---|---|---|---|
| Question 3: How do members of a stigmatized group (non-White women) respond to recruitment materials displaying racial/ethnic diversity, gender diversity, or both racial/ethnic and gender diversity? | Hypothesis 5: Non-White women will apply more to organizations that are presented as having any racial/ethnic diversity. | Data collection will continue until a minimum of 1036 eligible applications are submitted, which is expected to provide greater than 95% power to detect an OR = 1.72 (*d* = .30) main effect of racial/ethnic diversity. | Analysis 3: Logistic regression predicting the likelihood of applicant being a non-White woman from gender diversity condition, racial/ethnic diversity condition, interaction between conditions, and dummy-coded variable controlling for position applied for. See https://osf.io/8g65r/, lines 8-14 and 51-60. | 1) A main effect of racial/ethnic diversity, such that application rates are higher in conditions with racial/ethnic diversity, would suggest non-White female applicants respond positively to seeing ingroup racial/ethnic diversity reflected in organizational materials.<br><br>2) A main effect of gender diversity and a main effect of racial/ethnic diversity, such that racial/ethnic and gender diversity is associated with higher application rates, would suggest non-White female applicants respond positively to seeing both ingroup racial/ethnic and ingroup gender diversity reflected in organizational materials.<br><br>3) A main effect of gender diversity, such that application rates are higher in conditions with gender diversity, would suggest non-White female applicants respond |

| | | | | |
|---|---|---|---|---|
| | | | | positively to seeing ingroup gender diversity reflected in organizational materials.<br><br>4) Null results showing neither main effect of gender nor racial/ethnic diversity would suggest no evidence that our manipulations impacted application rates of non-White women. |
| Question 3: How do members of a stigmatized group (non-White women) respond to recruitment materials displaying racial/ethnic diversity, gender diversity, or both racial/ethnic and gender diversity? | Hypothesis 6: Non-White women will apply more to organizations that are presented as having any level of either racial/ethnic or gender diversity. | Data collection will continue until a minimum of 1524 eligible applications are submitted, which is expected to provide greater than 95% power to detect an OR = 2.07 (*d* = .40) interaction between racial/ethnic and gender diversity. | Analysis 3: Logistic regression predicting the likelihood of applicant being a non-White woman from gender diversity condition, racial/ethnic diversity condition, interaction between conditions, and dummy-coded variable controlling for position applied for. See https://osf.io/8g65r/, lines 8-14 and 51-60. | 1) See above for interpretations of main effects.<br><br>2) An interaction between gender and racial/ethnic diversity would suggest that, for non-White females, the impact of the gender or racial/ethnic diversity manipulation depends on the presence or absence of the other diversity manipulation.<br><br>3) Null results showing no gender by racial/ethnic interaction would suggest that, for non-White females, there was no evidence that the presence of the racial/ethnic diversity |

| | | | | |
|---|---|---|---|---|
| | | | | manipulation moderated the impact of the gender diversity manipulation (and vice versa). |
| Question 4: How do members of a non-stigmatized group (White men) respond to recruitment materials displaying racial/ethnic diversity, gender diversity, or both racial/ethnic and gender diversity? | Hypothesis 7: White men will apply less to organizations that are presented as having greater racial/ethnic diversity. | Data collection will continue until a minimum of 1036 eligible applications are submitted, which is expected to provide greater than 95% power to detect an OR = 0.58 (*d* = .30) main effect of racial/ethnic diversity. | Analysis 4: Logistic regression predicting the likelihood of applicant being a White man from gender diversity condition, racial/ethnic diversity condition, interaction between conditions, and dummy-coded variable controlling for position applied for. See https://osf.io/8g65r/, lines 8-14 and 18-27. | 1) A main effect of racial/ethnic diversity, such that application rates are lower in conditions with racial/ethnic diversity, would suggest White male applicants respond negatively to seeing outgroup racial/ethnic diversity reflected in organizational materials.<br><br>2) A main effect of racial/ethnic diversity, such that application rates are higher in conditions with racial/ethnic diversity, would suggest White male applicants respond positively to seeing outgroup racial/ethnic diversity reflected in organizational materials.<br><br>3) A main effect of gender diversity, such that application rates are lower in conditions with gender diversity, would suggest |

| | | | | |
|---|---|---|---|---|
| | | | | White male applicants respond negatively to seeing outgroup gender diversity reflected in organizational materials.<br><br>4) A main effect of gender diversity, such that application rates are higher in conditions with gender diversity, would suggest White male applicants respond positively to seeing outgroup racial/ethnic diversity reflected in organizational materials.<br><br>5) Null results showing neither main effect of gender nor racial/ethnic diversity would suggest no evidence that our manipulations impacted application rates of White men. |
| <u>Question 4:</u> How do members of a non-stigmatized group (White men) respond to recruitment materials displaying racial/ethnic diversity, gender | <u>Hypothesis 8:</u> White men will apply less to organizations that are presented as having any level of either racial/ethnic or gender diversity. | Data collection will continue until a minimum of 1524 eligible applications are submitted, which is expected to provide greater than 95% power to detect an OR = 0.48 (*d* = .40) | <u>Analysis 4:</u> Logistic regression predicting the likelihood of applicant being a White man from gender diversity condition, racial/ethnic diversity condition, interaction between conditions, and dummy-coded variable controlling for position applied for. See https://osf.io/8g65r/, | 1) See above for interpretations of main effects.<br><br>2) An interaction between gender and racial/ethnic diversity would suggest that, for White males, the impact of the gender or |

| | | | | |
|---|---|---|---|---|
| diversity, or both racial/ethnic and gender diversity? | | interaction between racial/ethnic and gender diversity. | lines 8-14 and 18-27. | racial/ethnic diversity manipulation on application rates depends on the presence or absence of the other diversity manipulation.<br><br>3) Null results showing no gender by racial/ethnic interaction would suggest that, for White males, there was no evidence that the presence of the racial/ethnic diversity manipulation moderated the impact of the gender diversity manipulation (and vice versa) on application rates. |
| <u>Question 5:</u> How is the quality of applicants from a stigmatized group (White women) impacted by recruitment materials displaying racial/ethnic diversity, gender diversity, or both racial/ethnic and gender diversity? | <u>Hypothesis 9:</u> The average quality of White female applicants will decrease when organizations are presented as having gender diversity. | Data collection will continue until there is an average of 42 eligible applicants per condition, which will provide 95% power for detecting a main effect of $d$ = .57 for either racial/ethnic or gender diversity. | <u>Analysis 5:</u> 2 (Racial/Ethnic Diversity) by 2 (Gender Diversity) between-subjects ANOVA on coded applicant quality. See https://osf.io/m2fd4/, lines 7-13 and 35-48. | 1) A main effect of gender diversity, such that application quality decreases in conditions with gender diversity, would suggest that recruitment materials displaying gender diversity increase organizational appeal among less-qualified White female applicants.<br><br>2) A main effect of gender diversity, such that application quality increases in conditions with |

| | |
|---|---|
| | gender diversity, would suggest that recruitment materials displaying gender diversity increase organizational appeal among more-qualified White female applicants.<br><br>3) An interaction between racial/ethnic and gender diversity would suggest that, for White females, the impact of the gender diversity manipulation on applicant quality depends on the presence or absence of racial/ethnic diversity also present in organizational materials.<br><br>4) Null results for main effects of the racial/ethnic and gender diversity manipulation would suggest no evidence that either manipulation impacted applicant quality of White females. A null result for the interaction would suggest that, for White females, there was no evidence that the impact of the racial/ethnic diversity manipulation on applicant |

| | | | | |
|---|---|---|---|---|
| | | | | quality was moderated by the presence of the gender diversity manipulation (and vice versa). |
| Question 6: How is the quality of applicants from a stigmatized group (non-White men) impacted by recruitment materials displaying racial/ethnic diversity, gender diversity, or both racial/ethnic and gender diversity? | Hypothesis 10: The average quality of non-White male applicants will decrease when organizations are presented as having greater racial/ethnic diversity. | Data collection will continue until there is an average of 74 eligible applicants per condition, which will provide 95% power for detecting a main effect of $d$ = .42 for either racial/ethnic or gender diversity. | Analysis 6: 2 (Racial/Ethnic Diversity) by 2 (Gender Diversity) between-subjects ANOVA on coded applicant quality. See https://osf.io/m2fd4/, lines 7-13 and 52-65. | 1) A main effect of racial/ethnic diversity, such that application quality decreases in conditions with racial/ethnic diversity, would suggest that recruitment materials displaying racial/ethnic diversity increase organizational appeal among less-qualified non-White male applicants.<br>2) A main effect of racial/ethnic diversity, such that application quality increases in conditions with racial/ethnic diversity, would suggest that recruitment materials displaying racial/ethnic diversity increase organizational appeal among more-qualified non-White male applicants.<br>3) An interaction between racial/ethnic and gender diversity would suggest |

| | | | | |
|---|---|---|---|---|
| | | | | that, for non-White males, the impact of the racial/ethnic diversity manipulation on applicant quality depends on the presence or absence of gender diversity also present in organizational materials.<br><br>4) Null results for main effects of the racial/ethnic and gender diversity manipulation would suggest no evidence that either manipulation impacted applicant quality of non-White males. A null result for the interaction would suggest that, for non-White males, there was no evidence that the impact of the racial/ethnic diversity manipulation on applicant quality was moderated by the presence of the gender diversity manipulation (and vice versa). |
| <u>Question 7:</u> How is the quality of applicants from a stigmatized group (non-White women) | <u>Hypothesis 11:</u> The average quality of non-White female applicants will | Data collection will continue until there is an average of 37 eligible applicants per condition, which will | <u>Analysis 7:</u> 2 (Racial/Ethnic Diversity) by 2 (Gender Diversity) between-subjects ANOVA on coded applicant quality. See | 1) A main effect of racial/ethnic diversity, such that application quality decreases in conditions with racial/ethnic diversity, |

| | | | | |
|---|---|---|---|---|
| impacted by recruitment materials displaying racial/ethnic diversity, gender diversity, or both racial/ethnic and gender diversity? | decrease when organizations are presented as having greater racial/ethnic diversity. | provide 95% power for detecting a main effect of *d* = .60 for either racial/ethnic or gender diversity | https://osf.io/m2fd4/, lines 7-13 and 69-82. | would suggest that recruitment materials displaying racial/ethnic diversity increase organizational appeal among less-qualified non-White female applicants.<br><br>2) A main effect of racial/ethnic diversity, such that application quality increases in conditions with racial/ethnic diversity, would suggest that recruitment materials displaying racial/ethnic diversity increase organizational appeal among more-qualified non-White female applicants.<br><br>3) A main effect of gender diversity, such that application quality decreases in conditions with gender diversity, would suggest that recruitment materials displaying gender diversity increase organizational appeal among less-qualified non-White female applicants.<br><br>4) A main effect of gender diversity, such that |

| | | | | |
|---|---|---|---|---|
| | | | | application quality increases in conditions with gender diversity, would suggest that recruitment materials displaying gender diversity increase organizational appeal among more-qualified non-White female applicants.<br><br>5) An interaction between racial/ethnic and gender diversity would suggest that, for non-White females, the impact of the racial/ethnic or gender diversity manipulation on applicant quality depends on the presence or absence of the other diversity manipulation also present in the materials.<br><br>6) Null results for main effects of the racial/ethnic and gender diversity manipulation would suggest no evidence that either manipulation impacted applicant quality of non-White females. A null result for the interaction would suggest that, for non-White females, there was no |

| | | | | |
|---|---|---|---|---|
| | | | | evidence that the impact of the racial/ethnic diversity manipulation on applicant quality was moderated by the presence of the gender diversity manipulation (and vice versa). |
| Question 8: How is the quality of applicants from a non-stigmatized group (White men) impacted by recruitment materials displaying racial/ethnic diversity, gender diversity, or both racial/ethnic and gender diversity? | Hypothesis 12: The average quality of White male applicants will increase when organizations are presented as having greater racial/ethnic or gender diversity. | Coders will rate applicants once there is at least an average of 75 eligible applicants per condition, which will provide 95% power for detecting a main effect of $d$ = .42 for either racial/ethnic or gender diversity. | Analysis 8: 2 (Racial/Ethnic Diversity) by 2 (Gender Diversity) between-subjects ANOVA on coded applicant quality. See https://osf.io/m2fd4/, lines 7-13 and 18-31. | 1) A main effect of racial/ethnic diversity, such that application quality decreases in conditions with racial/ethnic diversity, would suggest that recruitment materials displaying racial/ethnic diversity increase organizational appeal among less-qualified White male applicants.<br><br>2) A main effect of racial/ethnic diversity, such that application quality increases in conditions with racial/ethnic diversity, would suggest that recruitment materials displaying racial/ethnic diversity increase organizational appeal among more-qualified White male applicants.<br><br>3) A main effect of gender |

| | | | |
|---|---|---|---|
| | | | diversity, such that application quality decreases in conditions with gender diversity, would suggest that recruitment materials displaying gender diversity increase organizational appeal among less-qualified White male applicants.<br><br>4) A main effect of gender diversity, such that application quality increases in conditions with gender diversity, would suggest that recruitment materials displaying gender diversity increase organizational appeal among more-qualified White male applicants.<br><br>5) An interaction between racial/ethnic and gender diversity would suggest that, for White males, the impact of the racial/ethnic or gender diversity manipulation on applicant quality depends on the presence or absence of the other diversity manipulation also present in the |

| | | | | |
|---|---|---|---|---|
| | | | | materials.<br><br>6) Null results for main effects of the racial/ethnic and gender diversity manipulation would suggest no evidence that either manipulation impacted applicant quality of White males. A null result for the interaction would suggest that, for White males, there was no evidence that the impact of the racial/ethnic diversity manipulation on applicant quality was moderated by the presence of the gender diversity manipulation (and vice versa). |
| Question 9: How is the overall quantity of applicants to an organization impacted by recruitment materials displaying racial/ethnic diversity, gender diversity, or both racial/ethnic and gender diversity? | Hypothesis 13: Overall rate of application submission from website visitors will increase for organizations displaying racial/ethnic or gender diversity. | Data collection will continue until there are 1036 eligible applications submitted. Assuming 3% of website visitors submit applications, this sample size provides 95% power to detect OR = 1.42 (*d* = .19) for a main effect of racial/ethnic diversity, gender diversity, or an | Analysis 9: Logistic regression predicting the likelihood of website visitor submitting an application from gender diversity condition, racial/ethnic diversity condition, and interaction between conditions. See https://osf.io/2dyx9/, lines 8-14 and 24-28. | 1) A main effect of racial/ethnic diversity, such that applications increase in conditions with racial/ethnic diversity, would suggest that website visitors were more interested in applying to organizations displaying racial/ethnic diversity in recruitment materials.<br><br>2) A main effect of racial/ethnic diversity, such |

| | | | |
|---|---|---|---|
| | interaction. | | that applications decrease in conditions with racial/ethnic diversity, would suggest that website visitors were less interested in applying to organizations displaying racial/ethnic diversity in recruitment materials.<br><br>3) A main effect of gender diversity, such that applications increase in conditions with gender diversity, would suggest that website visitors were more interested in applying to organizations displaying gender diversity in recruitment materials.<br><br>4) A main effect of gender diversity, such that applications decrease in conditions with gender diversity, would suggest that website visitors were less interested in applying to organizations displaying gender diversity in recruitment materials.<br><br>5) An interaction between racial/ethnic and gender diversity would suggest that |

| | | | | |
|---|---|---|---|---|
| | | | | the impact of the racial/ethnic or gender diversity manipulation on the number of applications submitted depends on the presence or absence of the other diversity manipulation also present in the materials.<br><br>6) Null results for main effects of the racial/ethnic and gender diversity manipulation would suggest no evidence that either manipulation impacted number of applications submitted. A null result for the interaction would suggest no evidence that the impact of the racial/ethnic diversity manipulation on the number of applications was moderated by the presence of the gender diversity manipulation (and vice versa). |
| <u>Question 10:</u> How is the overall quantity of application started impacted by recruitment materials displaying | <u>Hypothesis 14:</u> Overall rate of applications started from website visitors will increase for | Data collection will continue until there are 1036 eligible applications submitted. Assuming 4% of website visitors start an | <u>Analysis 10:</u> Logistic regression predicting the likelihood of website visitor starting an application from gender diversity condition, racial/ethnic diversity condition, and | 1) A main effect of racial/ethnic diversity, such that applications increase in conditions with racial/ethnic diversity, would suggest that website |

| | | | | |
|---|---|---|---|---|
| racial/ethnic diversity, gender diversity, or both racial/ethnic and gender diversity? | organizations displaying racial/ethnic diversity or gender diversity. | application, this sample size provides 95% power to detect OR = 1.31 (*d* = .15) for a main effect of racial/ethnic diversity, gender diversity, or an interaction. | interaction between conditions. See https://osf.io/2dyx9/, lines 8-14 and 18-22. | visitors were more interested in starting applications to organizations displaying racial/ethnic diversity in recruitment materials.<br><br>2) A main effect of racial/ethnic diversity, such that applications decrease in conditions with racial/ethnic diversity, would suggest that website visitors were less interested in starting applications to organizations displaying racial/ethnic diversity in recruitment materials.<br><br>3) A main effect of gender diversity, such that applications increase in conditions with gender diversity, would suggest that website visitors were more interested in starting applications to organizations displaying gender diversity in recruitment materials.<br><br>4) A main effect of gender diversity, such that applications decrease in conditions with gender |

diversity, would suggest that website visitors were less interested in starting applications to organizations displaying gender diversity in recruitment materials.

5) An interaction between racial/ethnic and gender diversity would suggest that the impact of the racial/ethnic or gender diversity manipulation on the number of applications started depends on the presence or absence of the other diversity manipulation also present in the materials.

6) Null results for main effects of the racial/ethnic and gender diversity manipulation would suggest no evidence that either manipulation impacted number of applications started. A null result for the interaction would suggest no evidence that the impact of the racial/ethnic diversity manipulation was moderated by the presence

| | | |
|---|---|---|
| | | of the gender diversity manipulation (and vice versa). |

* - The threshold for determining statistical significance will be set at $\alpha = 0.05$.

**Supplementary Table 2. Resources Available in Online Repository**

| File Name | Description | Direct Link |
|---|---|---|
| Demographic Distributions, Condition Assignment and Maintenance | | |
| DemographicDistributionInfo | Summary of report for demographics of tech workers used to calculate anticipated sample distributions | https://osf.io/jtvnf/ |
| TechSectorDiversityDemographics | Source data for calculating anticipated sample distributions based on demographics | https://osf.io/xtucw/ |
| ConditionAssignmentMaintenance | Information on how participants are assigned to conditions across devices and IP addresses | https://osf.io/39fwt/ |
| Power Analyses | | |
| PowerAnalyses.AdditionalInfo | Background information for all subgroups used to calculate power analyses | https://osf.io/8asqp/ |
| Power.NonWhiteMen.Option1 | Power analysis for non-White men anticipating only a main effect of racial/ethnic diversity | https://osf.io/fdk2g/ |
| Power.NonWhiteMen.Option2 | Power analysis for non-White men anticipating an interaction between racial/ethnic and gender diversity | https://osf.io/fb3cv/ |
| Power.NonWhiteWomen.Option1 | Power analysis for non-White women anticipating only a main effect of racial/ethnic diversity | https://osf.io/pqd65/ |
| Power.NonWhiteWomen.Option2 | Power analysis for non-White women anticipating an interaction between racial/ethnic and gender diversity | https://osf.io/3hqmu/ |
| Power.WhiteMen.Option1 | Power analysis for White men anticipating only a main effect of racial/ethnic diversity | https://osf.io/a5ymp/ |
| Power.WhiteMen.Option2 | Power analysis for White men anticipating an interaction between racial/ethnic and gender diversity. | https://osf.io/uy5m8/ |
| Power.WhiteWomen.Option1 | Power analysis for White women anticipating only a main effect of gender diversity | https://osf.io/wzfyj/ |
| Power.WhiteWomen.Option2 | Power analysis for White women anticipating an interaction between racial/ethnic and gender diversity | https://osf.io/zy6gh/ |
| Sample Data and Analysis Scripts | | |
| Analysis.ApplicantDiversity.SampleData | Sample data for analysis concerning applicant diversity | https://osf.io/havq2/ |

| | | |
|---|---|---|
| Analysis.ApplicantDiversity.Syntax | Syntax for analysis concerning applicant diversity | https://osf.io/4hrjk/ |
| Analysis.ApplicantQuality.SampleData | Sample data for analysis concerning applicant quality | https://osf.io/8qsrg/ |
| Analysis.ApplicantQuality.Syntax | Syntax for analysis concerning applicant quality | https://osf.io/d5r2u/ |
| Analysis.TotalApplications.SampleData | Sample data for analysis concerning total applications started or completed | https://osf.io/kjfah/ |
| Analysis.TotalApplications.Syntax | Syntax for analysis concerning total applications started or completed | https://osf.io/9v4su/ |
| Analysis.Equivalence Tests | Syntax for equivalence tests to be run on any null results relevant for Hypotheses 1-14 specified in Table 1. | https://osf.io/qtdxk/ |
| <u>Experiment Data and Analysis Scripts</u> | | |
| Cleaning_Foodable_Data_For_Analyses_1_2.R | *R* script to clean data for Analysis 1 (applicant diversity) and Analysis 2 (applicant quality) | https://osf.io/dg8sz/ |
| Foodable_Analysis1_Syntax.sps | SPSS Syntax for Analysis 1, analysis concerning applicant diversity | https://osf.io/u3fv6/ |
| Foodable_Analysis2_Syntax.sps | SPSS Syntax for Analysis 2, analysis concerning applicant quality | https://osf.io/73kcq/ |
| Post_Exclusion_Data_Analyses_1_2.csv | Data for Analyses 1 and 2 with registered exclusions applied | https://osf.io/fwqv4/ |
| Pre_Exclusion_Data_Analyses_1_2.csv | Data for Analyses 1 and 2 <u>before</u> registered exclusions are applied (See Manuscript for exclusion details) | https://osf.io/qv5zb/ |
| Cleaning_Foodable_Data_For_Analyses_3.1-3.3.R | *R* script to clean data for Analysis 3 (job clicks, applications started, and applications submitted) | https://osf.io/ghjx8/ |
| Foodable_Analysis3_Syntax.sps | SPSS Syntax for Analysis 3, analysis of job clicks, applications started, and applications submitted | https://osf.io/tz9rh/ |
| Analysis3_Survey_Job_Info_Click_Step1.csv | Data for Analysis 3 Step 1, analysis concerning job information clicks. | https://osf.io/pf5g7/ |
| Analysis3_Survey_Opens_Step2.csv | Data for Analysis 3 Step 2, analysis concerning application survey starts. | https://osf.io/m3rwd/ |
| Analysis3_Survey_Submissions_Step3.csv | Data for Analysis 3 Step 3, analysis concerning application survey submissions. | https://osf.io/zcpng/ |

| | | |
|---|---|---|
| Foodable_Experiment_Data_Raw.xlsx | Source data for experiment, Foodable website activity | https://osf.io/4pvgq/ |
| Survey_Submissions_Only.xlsx | Dataset containing only survey submissions, and their associated demographics and exclusion indicators | https://osf.io/qjns2/ |
| Users_That_Bypassed_Website.csv | Data with user ids for participants that bypassed the treatment (website) and accessed the application directly | https://osf.io/ah5sv/ |
| Website Source Code | | |
| Website Source Code.zip | A zip file of source code and materials used to create the website. | https://osf.io/pf8am/ |
| Variable Key | | |
| Variable_Key.csv | A variable key for dataset: Foodable_Experiment_Data_Raw.xlsx | https://osf.io/6q5kj/ |
| Stage 1 Registered Report | | |
| Stage_1_Protocol.pdf | Stage 1 Registered Report Protocol | https://osf.io/unbc8/ |

**Supplementary Table 3. Analysis of Applicant Demographics, 30-second Cutoff**
Series of binary logistic regressions predicting applicant demographics from racial/ethnic diversity condition, gender diversity condition, and their interaction. OR = Odds ratio.

Outcome: Applicant is a White Man

| *Term* | *B (S.E)* | *p* | *OR [95% CI]* |
|---|---|---|---|
| Racial/Ethnic Diversity | .34 (.23) | .145 | 1.40 [.89, 2.19] |
| Gender/Ethnic Diversity | .13 (.25) | .604 | 1.14 [.70, 1.87] |
| Racial/Ethnic * Gender Diversity | -.40 (.34) | .236 | .67 [.35, 1.30] |

Outcome: Applicant is a Non-White Man

| *Term* | *B (S.E)* | *p* | *OR* |
|---|---|---|---|
| Racial/Ethnic Diversity | -.26 (.18) | .144 | .77 [.54, 1.09] |
| Gender/Ethnic Diversity | .13 (.19) | .485 | 1.14 [.79, 1.65] |
| Racial/Ethnic * Gender Diversity | .06 (.26) | .817 | 1.06 [.64, 1.76] |

Outcome: Applicant is a White Woman

| *Term* | *B (S.E)* | *P* | *OR* |
|---|---|---|---|
| Racial/Ethnic Diversity | -.02 (.26) | .937 | .98 [.59, 1.62] |
| Gender Diversity | -.52 (.31) | .093 | .60 [.33, 1.09] |
| Racial/Ethnic * Gender Diversity | .24 (.41) | .561 | 1.27 [.57, 2.81] |

Outcome: Applicant is a Non-White Woman

| *Term* | *B (S.E)* | *p* | *OR* |
|---|---|---|---|
| Racial/Ethnic Diversity | .09 (.20) | .667 | 1.09 [.74, 1.62] |
| Gender/Ethnic Diversity | .002 (.22) | .993 | 1.00 [.66, 1.53] |
| Racial/Ethnic * Gender Diversity | .12 (.29) | .686 | 1.12 [.64, 1.98] |

**Supplementary Table 4. Analysis of Applicant Quality, 30-second Cutoff**
Series of 2 (Racial/Ethnic Diversity) by 2 (Gender Diversity) ANOVAs for each demographic group.

White Men Applicants ($N$ = 177)

| *Term* | *F* | *P* | $\eta_p^2$ |
|---|---|---|---|
| Racial/Ethnic Diversity | 2.16 | .144 | .012 |
| Gender Diversity | .05 | .830 | <.001 |
| Racial/Ethnic * Gender Diversity | .78 | .378 | .004 |

Non-White Men Applicants ($N$ = 204)

| *Term* | *F* | *P* | $\eta_p^2$ |
|---|---|---|---|
| Racial/Ethnic Diversity | 2.19 | .140 | .011 |
| Gender Diversity | 1.34 | .249 | .007 |
| Racial/Ethnic * Gender Diversity | 4.93 | .027 | .024 |

White Women Applicants ($N$ = 107)

| *Term* | *F* | *p* | $\eta_p^2$ |
|---|---|---|---|
| Racial/Ethnic Diversity | .03 | .853 | <.001 |
| Gender Diversity | 1.73 | .191 | .017 |
| Racial/Ethnic * Gender Diversity | 2.31 | .132 | .022 |

Non-White Women Applicants ($N$ = 99)

| *Term* | *F* | *p* | $\eta_p^2$ |
|---|---|---|---|
| Racial/Ethnic Diversity | 4.34 | .040 | .044 |
| Gender Diversity | 3.20 | .077 | .033 |
| Racial/Ethnic * Gender Diversity | 2.14 | .147 | .022 |

**Supplementary Table 5. Analysis of Applicant Demographics, No Exclusions**

Series of binary logistic regressions predicting applicant demographics from racial/ethnic diversity condition, gender diversity condition, and their interaction. OR = Odds ratio.

| Outcome: Applicant is a White Man | | | |
|---|---|---|---|
| *Term* | *B (S.E)* | *p* | *OR [95% CI]* |
| Racial/Ethnic Diversity | .10 (.18) | .560 | 1.11 [.79, 1.56] |
| Gender Diversity | -.05 (.18) | .777 | .95 [.67, 1.36] |
| Racial/Ethnic * Gender Diversity | -.20 (.25) | .431 | .82 [.50, 1.35] |
| Outcome: Applicant is a Non-White Man | | | |
| *Term* | *B (S.E)* | *p* | *OR* |
| Racial/Ethnic Diversity | -.05 (.14) | .733 | .95 [.73, 1.25] |
| Gender Diversity | -.01 (.14) | .946 | .99 [.75, 1.30] |
| Racial/Ethnic * Gender Diversity | .10 (.20) | .608 | 1.11 [.75, 1.62] |
| Outcome: Applicant is a White Woman | | | |
| *Term* | *B (S.E)* | *p* | *OR* |
| Racial/Ethnic Diversity | .16 (.21) | .456 | 1.17 [.78, 1.76] |
| Gender Diversity | -.02 (.22) | .918 | .98 [.64, 1.50] |
| Racial/Ethnic * Gender Diversity | -.42 (.31) | .169 | .65 [.36, 1.20] |
| Outcome: Applicant is a Non-White Woman | | | |
| *Term* | *B (S.E)* | *p* | *OR* |
| Racial/Ethnic Diversity | -.11 (.16) | .476 | .89 [.65, 1.22] |
| Gender Diversity | .07 (.16) | .645 | 1.08 [.79, 1.46] |
| Racial/Ethnic * Gender Diversity | .18 (.22) | .411 | 1.20 [.78, 1.85] |

Series of binary logistic regressions predicting applicant demographics from racial/ethnic diversity condition, gender diversity condition, and their interaction. OR = Odds ratio.

**Supplementary Table 6. Analysis of Applicant Quality, No Exclusions**
Series of 2 (Racial/Ethnic Diversity) by 2 (Gender Diversity) ANOVAs for each demographic group.

| White Men Applicants ($N$ = 287) | | | |
|---|---|---|---|
| *Term* | $F$ | $P$ | $\eta_p^2$ |
| Racial/Ethnic Diversity | .19 | .661 | .001 |
| Gender Diversity | .08 | .776 | <.001 |
| Racial/Ethnic * Gender Diversity | .94 | .334 | .003 |
| Non-White Men Applicants ($N$ = 325) | | | |
| *Term* | $F$ | $P$ | $\eta_p^2$ |
| Racial/Ethnic Diversity | .72 | .396 | .002 |
| Gender/Ethnic Diversity | .28 | .600 | .001 |
| Racial/Ethnic * Gender Diversity | .24 | .624 | .001 |
| White Women Applicants ($N$ = 168) | | | |
| *Term* | $F$ | $p$ | $\eta_p^2$ |
| Racial/Ethnic Diversity | .30 | .586 | .002 |
| Gender Diversity | 1.43 | .234 | .009 |
| Racial/Ethnic * Gender Diversity | .46 | .500 | .003 |
| Non-White Women Applicants ($N$ = 159) | | | |
| *Term* | $F$ | $p$ | $\eta_p^2$ |
| Racial/Ethnic Diversity | 11.18 | .001 | .067 |
| Gender Diversity | 6.20 | .014 | .038 |
| Racial/Ethnic * Gender Diversity | 2.26 | .135 | .014 |

Series of 2 (Racial Diversity) by 2 (Gender Diversity) ANOVAs for each demographic group.

**Supplementary Table 7. Analysis of Gender Quality Effect**
Racial/Ethnic and gender diversity conditions were coded such that 1 = Diversity present and 0 = Diversity absent.

| Overall Applicant Quality (*N* = 902) | | | |
|---|---|---|---|
| *Term* | *F* | *p* | $\eta_p^2$ |
| Participant Race/Ethnicity | <.001 | .990 | <.001 |
| Participant Gender | 41.41 | <.001 | .044 |
| Racial/Ethnic Diversity Cond | .44 | .508 | <.001 |
| Gender Diversity Cond | 4.06 | .044 | .004 |
| Part. Race/Ethnicity * Part. Gender | .62 | .433 | .001 |
| Part. Race/Ethnicity * Racial/Ethnicity Cond. | 3.85 | .050 | .004 |
| Part. Race/Ethnicity * Gender Cond. | .65 | .421 | .001 |
| Part. Gender * Racial/Ethnicity Cond. | 4.21 | .041 | .005 |
| Part. Gender * Gender Cond. | 2.74 | .099 | .003 |
| Racial/Ethnicity Cond. * Gender Cond. | .49 | .486 | .001 |
| Part. Race/Ethnicity * Part. Gender * Racial/Ethnicity Cond. | 3.89 | .049 | .004 |
| Part. Race/Ethnicity * Part. Gender * Gender Cond. | .04 | .847 | <.001 |
| Part. Race/Ethnicity * Racial/Ethnicity Cond. * Gender Cond. | .30 | .584 | <.001 |
| Part. Gender * Racial/Ethnicity Cond. * Gender Cond. | .05 | .824 | <.001 |
| Part. Race/Ethnicity * Part Gender. * Racial/Ethnicity Cond. * Gender Cond. | 3.54 | .060 | .004 |

**Supplementary Table 8. Analysis of Applicant Demographics, Excluding Duplicate IP Visits**
Series of binary logistic regressions predicting applicant demographics from racial/ethnic diversity condition, gender diversity condition, and their interaction. OR = Odds ratio.

Outcome: Applicant is a White Man

| *Term* | *B (S.E)* | *p* | *OR [95% CI]* |
|---|---|---|---|
| Racial/Ethnic Diversity | .12 (.19) | .525 | 1.13 [.78, 1.62] |
| Gender Diversity | -.04 (.19) | .854 | .97 [.66, 1.41] |
| Racial/Ethnic * Gender Diversity | -.23 (.27) | .396 | .80 [.47, 1.35] |

Outcome: Applicant is a Non-White Man

| *Term* | *B (S.E)* | *p* | *OR* |
|---|---|---|---|
| Racial/Ethnic Diversity | -.12 (.15) | .408 | .88 [.66, 1.18] |
| Gender Diversity | -.04 (.15) | .804 | .96 [.72, 1.29] |
| Racial/Ethnic * Gender Diversity | .18 (.21) | .384 | 1.20 [.80, 1.81] |

Outcome: Applicant is a White Woman

| *Term* | *B (S.E)* | *p* | *OR* |
|---|---|---|---|
| Racial/Ethnic Diversity | .18 (.22) | .409 | 1.20 [.78, 1.83] |
| Gender Diversity | -.09 (.23) | .683 | .91 [.58, 1.43] |
| Racial/Ethnic * Gender Diversity | -.31 (.32) | .337 | .74 [.39, 1.38] |

Outcome: Applicant is a Non-White Woman

| *Term* | *B (S.E)* | *p* | *OR* |
|---|---|---|---|
| Racial/Ethnic Diversity | -.05 (.17) | .790 | .96 [.69, 1.33] |
| Gender Diversity | .12 (.17) | .465 | 1.13 [.82, 1.56] |
| Racial/Ethnic * Gender Diversity | .11 (.23) | .643 | 1.11 [.71, 1.76] |

**Supplementary Table 9. Analysis of Applicant Quality, Excluding Duplicate IP Visits**
Series of 2 (Racial/Ethnic Diversity) by 2 (Gender Diversity) ANOVAs for each demographic group.

White Men Applicants ($N$ = 273)

| *Term* | *F* | *P* | $\eta_p^2$ |
|---|---|---|---|
| Racial/Ethnic Diversity | .29 | .590 | .001 |
| Gender Diversity | .04 | .853 | <.001 |
| Racial/Ethnic * Gender Diversity | .78 | .378 | .003 |

Non-White Men Applicants ($N$ = 308)

| *Term* | *F* | *P* | $\eta_p^2$ |
|---|---|---|---|
| Racial/Ethnic Diversity | 1.07 | .302 | .003 |
| Gender Diversity | .42 | .516 | .001 |
| Racial/Ethnic * Gender Diversity | .35 | .556 | .001 |

White Women Applicants ($N$ = 164)

| *Term* | *F* | *p* | $\eta_p^2$ |
|---|---|---|---|
| Racial/Ethnic Diversity | .21 | .652 | .001 |
| Gender Diversity | 1.82 | .179 | .011 |
| Racial/Ethnic * Gender Diversity | .36 | .548 | .002 |

Non-White Women Applicants ($N$ = 154)

| *Term* | *F* | *P* | $\eta_p^2$ |
|---|---|---|---|
| Racial/Ethnic Diversity | 9.28 | .003 | .058 |
| Gender Diversity | 5.92 | .016 | .038 |
| Racial/Ethnic * Gender Diversity | 3.20 | .076 | .021 |

**Supplementary Table 10. Analysis of Applications Started or Submitted, Limiting to One Row Per Participant (No Repeat Actions)**
Series of binary logistic regressions predicting application-related behaviors from racial/ethnic diversity condition, gender diversity condition, and their interaction. OR = Odds ratio.

Outcome: Submitting an Application

| *Term* | *B (S.E)* | *p* | *OR [95% CI]* |
|---|---|---|---|
| Racial/Ethnic Diversity | -.43 (.08) | <.001 | .65 [.56, .76] |
| Gender Diversity | -.28 (.08) | <.001 | .75 [.65, .88] |
| Racial/Ethnic * Gender Diversity | .78 (.11) | <.001 | 2.19 [1.76, 2.72] |

Outcome: Starting an Application

| *Term* | *B (S.E)* | *p* | *OR [95% CI]* |
|---|---|---|---|
| Racial/Ethnic Diversity | -.40 (.05) | <.001 | .67 [.61, .74] |
| Gender Diversity | -.27 (.05) | <.001 | .77 [.70, .85] |
| Racial/Ethnic * Gender Diversity | .72 (.07) | <.001 | 2.05 [1.79, 2.35] |

Outcome: Clicking on Job Advertisement

| *Term* | *B (S.E)* | *p* | *OR [95% CI]* |
|---|---|---|---|
| Racial/Ethnic Diversity | -.43 (.04) | <.001 | .65 [.60, .70] |
| Gender Diversity | -.35 (.04) | <.001 | .70 [.65, .76] |
| Racial/Ethnic * Gender Diversity | .78 (.06) | <.001 | 2.17 [1.94, 2.43] |

## SUPPLEMENTARY FIGURES

**Supplementary Figure 1. Welcome Screen**
Supplementary Figure 1 shows the welcome screen participants viewed when visiting the website. Photo by Joseph Gonzalez on Unsplash.

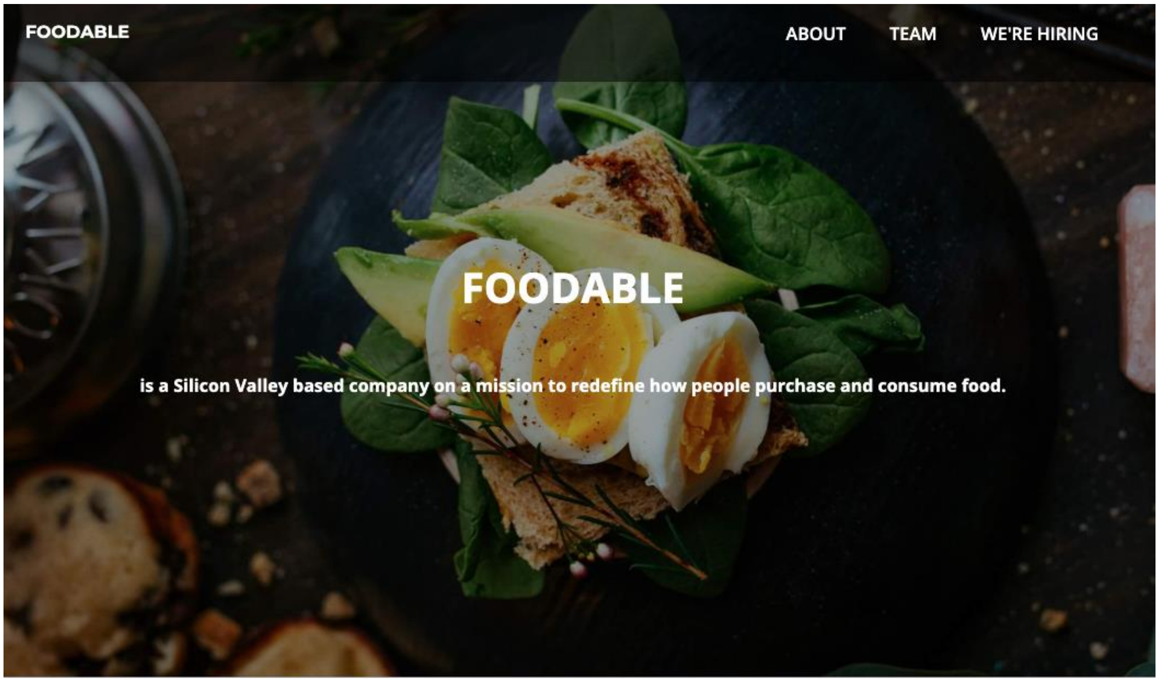

**Supplementary Figure 2. ‘About Us’ Section**

Supplementary Figure 2 shows the ‘About Us’ section of the website.

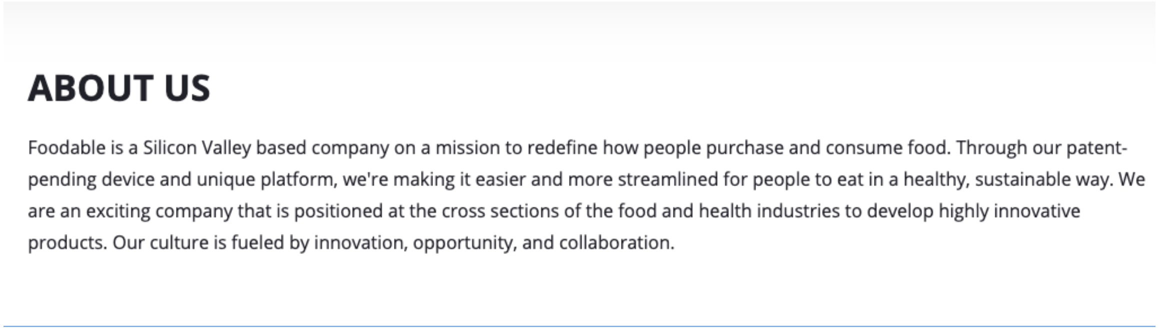

## ABOUT US

Foodable is a Silicon Valley based company on a mission to redefine how people purchase and consume food. Through our patent-pending device and unique platform, we're making it easier and more streamlined for people to eat in a healthy, sustainable way. We are an exciting company that is positioned at the cross sections of the food and health industries to develop highly innovative products. Our culture is fueled by innovation, opportunity, and collaboration.

**Supplementary Figure 3. Sample 'Team' Section**

Supplementary Figure 3 shows an example of the 'Team' section participants viewed when visiting the website. The team was varied according to condition (See Figure 1). The credit line for this figure can be found at the end of this document.

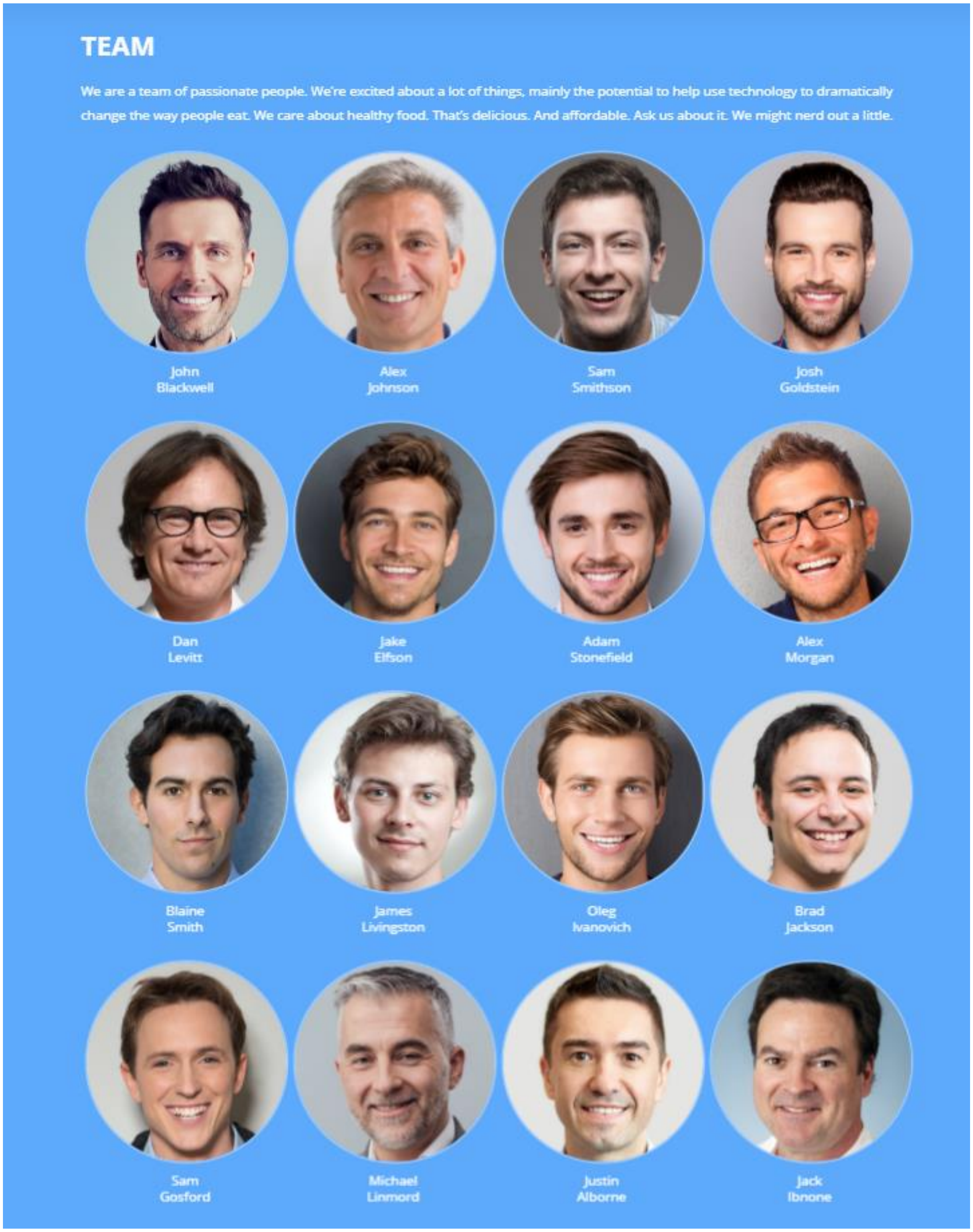

**Supplementary Figure 4. ‘We’re Hiring’ Section**
Supplementary Figure 4 shows the bottom of the webpage, the ‘We’re Hiring' section. The credit line for this figure can be found at the end of this document.

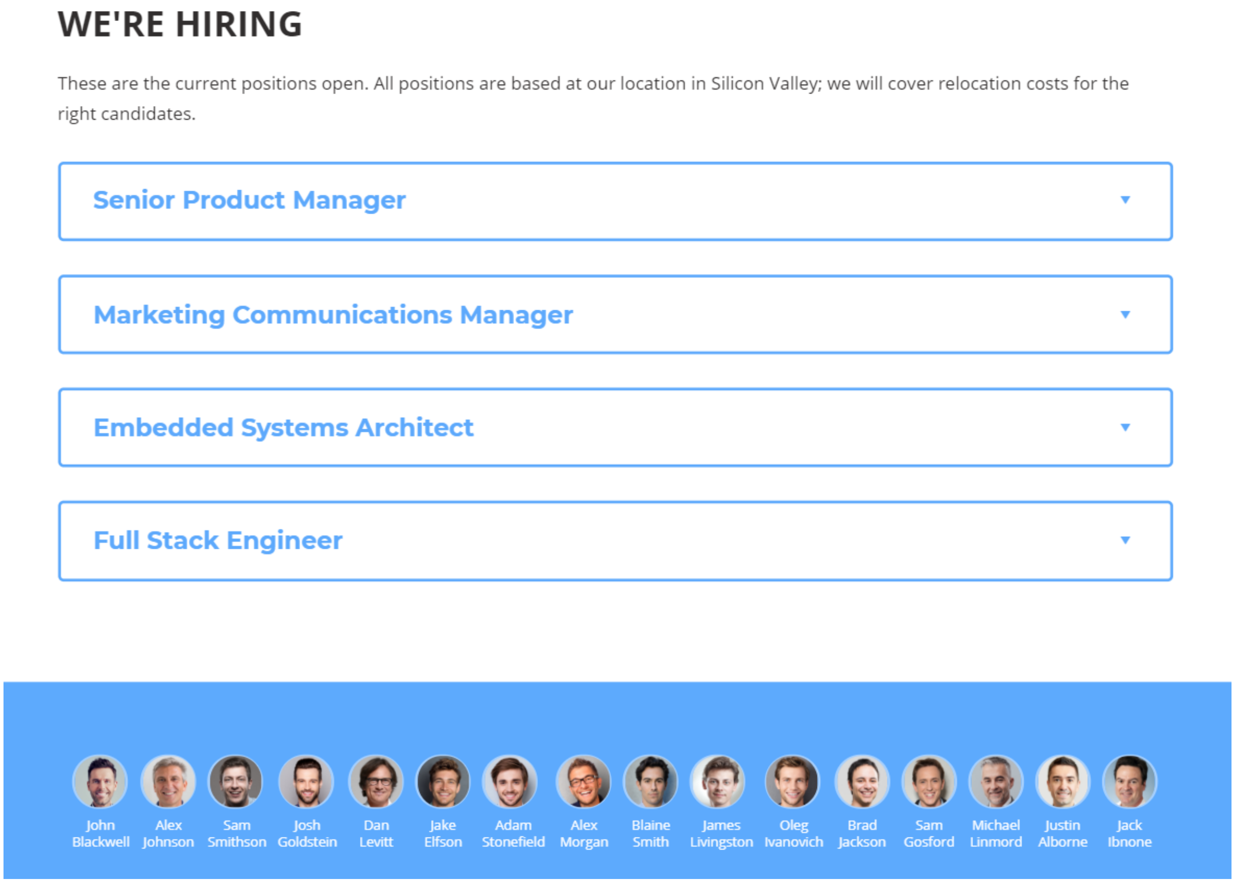

**Supplementary Figure 5. Senior Product Manager**

Supplementary Figure 5 shows the job description of the Senior Product Manager position as displayed on the website.

Senior Product Manager

## ROLE

The Sr. Product Marketing Manager will develop an intimate understanding of the market and which use-cases and segments to prioritize. You will be responsible for creating a product roadmap that addresses these segments and identifying the right feature set for each release.

## RESPONSIBILITIES

- Identify and define key use cases and market segments to pursue for new products and to grow our current offerings in.
- Develop the product roadmap and lead the planning, definition and prioritization of features for future product releases by conducting market research.
- Help define the go-to-market strategy for each product release.
- Identify and evaluate potential partnerships that support the go-to-market strategy.
- Develop the core value proposition for the product and it's respective positioning and messaging.
- Be an expert with respect to competitive offerings in order to deliver products recognized as thought leading in the industry.
- Define business models for product offerings and set the corresponding pricing strategy.
- Work with finance to produce revenue forecasts
- Define and analyze metrics that inform the success of your product.

## QUALIFICATIONS

- Entrepreneurial spirit with a strong desire to make the world a better place.
- A minimum of a Bachelor's degree in Marketing, Business or a related field is required. An MBA is preferred but not required.
- A minimum of 5 years of product management experience is required.
- Demonstrated success in defining and launching new products that include a hardware component.
- Proven ability to influence cross-functional teams without formal authority.
- Experience managing and prioritizing multiple projects is required.
- Excellent communication skills (written and oral) are required.

## BENEFITS

We offer a competitive benefits package including vacation, holidays, 401(k), medical, and dental. Food and snacks are provided on our premises.

APPLY HERE

## Supplementary Figure 6. Marketing Communications Manager

Supplementary Figure 6 shows the job description of the Marketing Communications Manager position.

**Marketing Communications Manager**

**ROLE**
The Marketing Communications Manager is a communication specialist responsible for executing a wide range of informational materials for internal and external audiences. The Marketing Communications Manager will manage the development and execution of go-to-market strategies and campaigns that drive awareness, engagement and adoption of our solutions.

**RESPONSIBILITIES**

- Develop and deliver impactful, strategic marketing communications programs and campaigns in support of business goals, such as brand differentiation, market awareness, demand generation and sales enablement with prospects as well as existing customers.
- Work cross-functionally with marketing program managers, product marketing and creative and web services teams to facilitate successful execution of new product launches.
- Partner with the Business Unit to develop multi-channel and multi-media marketing content in support of marketing objectives (including but not limited to press releases, website content, product collateral including datasheets, brochures, white papers and success stories, webinars, videos, contributed editorial articles and advertisements).
- Use digital marketing technology and techniques, including marketing automation and customer engagement platforms, search engine marketing (SEM), search engine optimization (SEO) and social media tools to promote and market products and services.
- Develop and implement methods of measuring marketing effectiveness and ROI.
- Provide leadership in managing events of various sizes, working cross-functionally with marketing program management, product marketing and an events management team.

**QUALIFICATIONS**

- Entrepreneurial spirit with a strong desire to make the world a better place.
- A minimum of a Bachelor's degree in Marketing, Business or a related field is required.
- A minimum of 3 years of marketing experience is required, including experience in digital marketing.
- Experience managing and prioritizing multiple projects is required.
- Excellent communication skills (written and oral) are required.

**BENEFITS**
We offer a competitive benefits package including vacation, holidays, 401(k), medical, and dental. Food and snacks are provided on our premises.

## Supplementary Figure 7. Embedded Systems Architect

Supplementary Figure 7 shows the job description of the Embedded Systems Architect position.

**Embedded Systems Architect**

**ROLE**

As the Embedded Systems Architect, you are the key person in driving various aspects of our hardware designs that range from small durable form-factors such as mobile sensors to medical grade equipment. You will collaborate with many internal cross-functional teams from compliance and certification to service software engineering teams to test and QA. In order for you to succeed, strong communication skills and a strong customer oriented passion are a must. Our team values open collaboration and positive work environments.

**RESPONSIBILITIES**

- Design hardware for mobile devices, commercial food grade products and medical devices.
- Contribute to cross functional designs including enclosures, pcb layout and efficient RTOS software.
- Drive decisions for quality designs that balance, manufacturability, technology longevity and cost.
- Actively participate in design reviews.
- Support products through their lifecycle.

**QUALIFICATIONS**

- Degree in Electrical Engineering, Computer Science or related field experience with 5+ years in consumer facing embedded systems required. Masters Degree or PhD preferred.
- Experience with sensor design and radio technologies is a must.
- Experience with Medical and low-cost small form factor designs are a strong plus.
- Solid communication skills coupled with technical document writing are a must.
- Solid track record of designing and building low-cost manufacturable devices is required.

**BENEFITS**

We offer a competitive benefits package including vacation, holidays, 401(k), medical, and dental. Food and snacks are provided on our premises.

## Supplementary Figure 8. Full Stack Engineer

Supplementary Figure 8 shows the job description of the Full Stack Engineer position.

**Full Stack Engineer**

**ROLE**
As the Senior Full Stack Engineer, you are empowered to build our services from backend databases all the way to mobile and web-clients. Since our team is small and growing, picking the right technology choices for our service oriented architecture that can scale to a variety of clients will be your primary goal. Fast, Scalable and Secure are the key.

**RESPONSIBILITIES**

- Build robust fault tolerant, scalable, secure and efficient services end to end.
- Willingness to dive into the client side code for minor and major features.
- Collaborate closely with cross functional teams from hardware, Site and Reliability and frontend engineers.
- Comfortable working in Unix environments.
- Participate in design reviews.
- Drive design decisions for technology choices across the stack.
- Integrate and develop performance analytics. Design products that alert us to problems before the customer knows.

**QUALIFICATIONS**

- Degree in Computer Science, Computer Engineering or related field experience with 5+ years in full stack development is required. Masters Degree or PhD preferred.
- Expertise in Javascript, HTML and MVC technologies Rails, Django, Twisted, etc.
- Some experience in Java, Go or Python for backend services required.
- Strong understanding of service oriented architecture and how design decisions impact various clients is a must.
- Proven ability to learn new technologies, whether backend, frontend or databases to find the right solution for the team.
- Experience with performance analysis is a plus.
- Developing HIPAA compliant systems is a strong plus.

**BENEFITS**
We offer a competitive benefits package including vacation, holidays, 401(k), medical, and dental. Food and snacks are provided on our premises.

## Supplementary Figure 9. Web Developer

Supplementary Figure 9 shows the job description of the Web Developer position.

**Web Developer**

**ROLE**
This role is particularly well-suited for someone who values creating high quality web pages and user experiences, as well as someone who has a strong sense of ownership of their product.

**RESPONSIBILITIES**

- Build out full-stack website features to help drive growth and engagement
- Maintain and grow our front facing web application
- Help grow our business through quick experimentation and feature development
- Data analytics and instrumentation using Google Analytics
- Publish content to our website using CMS

**QUALIFICATIONS**

- A demonstrated ability to produce high-quality code
- Computer Science background and understanding of algorithms and data structures.
- Experience with modern software development including pull requests, continuous integration/deployment, unit testing, and agile methodologies.
- Experience with cloud technologies.
- Experience writing SQL and tuning relational database queries.
- Proficiency in creating integrations with external APIs.
- Experience in front-end technology and framework such as HTML, CSS, JavaScript, BackboneJS, ReactJS or similar.
- Familiar with development concepts such as Dependency Injection, Mocking/Test Doubles, Object Relational Mappers.
- Appreciation for testing and the ability to write specs.
- Experience working and collaborating with designers.
- Experience with data analytics tools like Google Analytics, or similar, is preferred.
- Strong prioritization skills

**BENEFITS**
We offer a competitive benefits package including vacation, holidays, 401(k), medical, and dental. Food and snacks are provided on our premises.

## Supplementary Figure 10. Account Manager

Supplementary Figure 10 shows the job description of the Account Manager position.

**Account Manager**

**ROLE**
The Account Manager will be in charge of maintaining current client relationships, and develop new business from existing accounts.

**RESPONSIBILITIES**

- Deliver top-notch customer service by providing clients with prompt responses and helping meet their needs.
- Identify new business opportunities within existing customer base.
- Provide high level product training for customers.
- Manage growing book of customers.
- Understand the functions of our product and mission and be able to articulate our key features to potential clients.
- Stay up to date on relevant products and technology.
- Build and maintain strong client relationships.
- Prepare and submit monthly account forecasts.

**QUALIFICATIONS**

- Results-driven focus to satisfy existing clients.
- A minimum of a Bachelor's degree is required.
- Familiarity selling to large corporations across different industries.
- Knowledge of accounting and financial concepts is preferred.
- Proven history of success in technology or a related field.
- Working knowledge of Microsoft Office.

**BENEFITS**
We offer a competitive benefits package including vacation, holidays, 401(k), medical, and dental. Food and snacks are provided on our premises.

## Supplementary Figure 11. Business Analyst

Supplementary Figure 11 shows the job description of the Business Analyst position.

**Business Analyst**

**ROLE**
The Business Analyst will play a vital role in coordinating between the business team and other teams throughout Foodable.

**RESPONSIBILITIES**

- Analyze and synthesize information provided by stakeholders.
- Lead ongoing reviews of business processes and developing optimization strategies.
- Conduct interviews and independent research to design solutions for business problems.
- Stay up to date on latest advancements to modernize Foodable systems.
- Document, track, and resolve issues related to data quality.
- Remain available daily for questions from other project members.
- Work closely with clients, technicians and managerial staff.
- Lead or facilitate business requirement workshops.
- Effectively communicate insights to team members and management.

**QUALIFICATIONS**

- Detail-oriented, analytical mindset to find issues and identify solutions.
- A Bachelor’s degree in business or a related field.
- Ability to influence without direct authority.
- Adaptable to changes in a fast-paced environment.
- Working knowledge of Microsoft Office
- Exceptional analytical and conceptual thinking skills
- Excellent documentation skills

**BENEFITS**
We offer a competitive benefits package including vacation, holidays, 401(k), medical, and dental. Food and snacks are provided on our premises.

## Supplementary Figure 12. Copywriter

Supplementary Figure 12 shows the job description of the Copywriter position.

**Copywriter**

**ROLE**
Your writing will shape user interfaces, enhance UX, define product value propositions, and clarify in-app messaging. It will also support marketing initiatives.

**RESPONSIBILITIES**

- Take complicated product challenges and translate them into compelling, clear brand stories that are understandable and interesting.
- Support writing needs across multiple projects, including upcoming campaign work, product messaging, social and digital copy and pitch decks.
- Collaborate with the visual design team, the digital/social team, and the product marketing teams to express our voice in a variety of projects.
- Be comfortable working as the lead writer, with direction from Product Managers, to translate strategy into compelling copy in both short and long form.
- Combine a passion for copywriting and copyediting with a drive to create communication that makes readers take action, using clear and memorable consumer-facing language.

**QUALIFICATIONS**

- Proven advanced writing skills
- Strong editing and proofreading skills.
- The ability to work quickly and meet tight deadlines on a multitude of concurrent tasks.
- The ability to work well both independently and in a team. Experience managing and prioritizing multiple projects is required.
- The ability (and desire) to ideate on things like campaign themes, ad concepts, and product names.

**BENEFITS**
We offer a competitive benefits package including vacation, holidays, 401(k), medical, and dental. Food and snacks are provided on our premises.

## Supplementary Figure 13. Recruiter

Supplementary Figure 13 shows the job description of the Recruiter position.

**ROLE**

The Recruiter role will be in charge of coordinating the hiring aspects of the company. You will be responsible for using various resources and platforms to locate the right candidates for our growing team.

**RESPONSIBILITIES**

- Manage full-cycle employee recruitment across multiple teams.
- Generate a diverse candidate population using methods like including job boards, membership organizations, and personal networking.
- Create efficient and effective recruitment strategies.
- Conduct phone interviews with applicants to ensure candidates meet minimum qualifications.
- Screen candidate applications to ensure qualifications.
- Research new tools for effective recruitment and best practices.
- Provide information to potential applicants about the Foodable mission and team.
- Communicate status and progress on filling open positions.
- Ensure a smooth application process for candidates.

**QUALIFICATIONS**

- Motivated attitudes to go the extra mile to find the right candidate.
- Some prior experience in recruitment is preferred.
- Demonstrated success in facilitating with the screening and hiring of potential candidates.
- Proven ability to handle sensitive and confidential information.
- Experience managing multiple ongoing projects is required.
- Excellent communication skills (written and oral) are required.

**BENEFITS**

We offer a competitive benefits package including vacation, holidays, 401(k), medical, and dental. Food and snacks are provided on our premises.

**Supplementary Figure 14. Application Portal, Part 1**
Supplementary Figure 14 shows part 1 of the application in the website application portal.

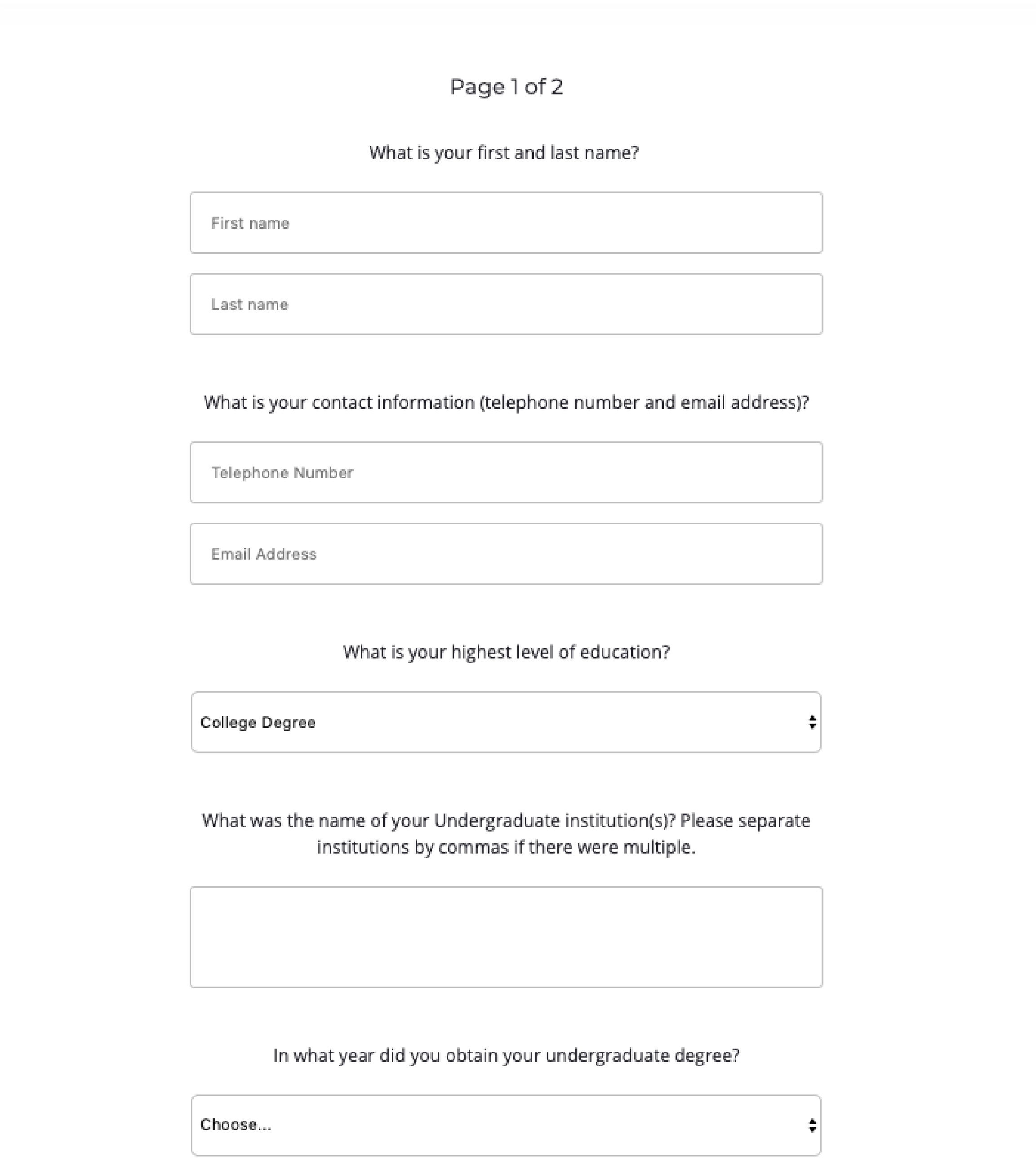
Page 1 of 2

What is your first and last name?

First name

Last name

What is your contact information (telephone number and email address)?

Telephone Number

Email Address

What is your highest level of education?

College Degree

What was the name of your Undergraduate institution(s)? Please separate institutions by commas if there were multiple.

In what year did you obtain your undergraduate degree?

Choose...

**Supplementary Figure 15. Application Portal, Part 2**
Supplementary Figure 15 shows part 2 of the application in the website application portal.

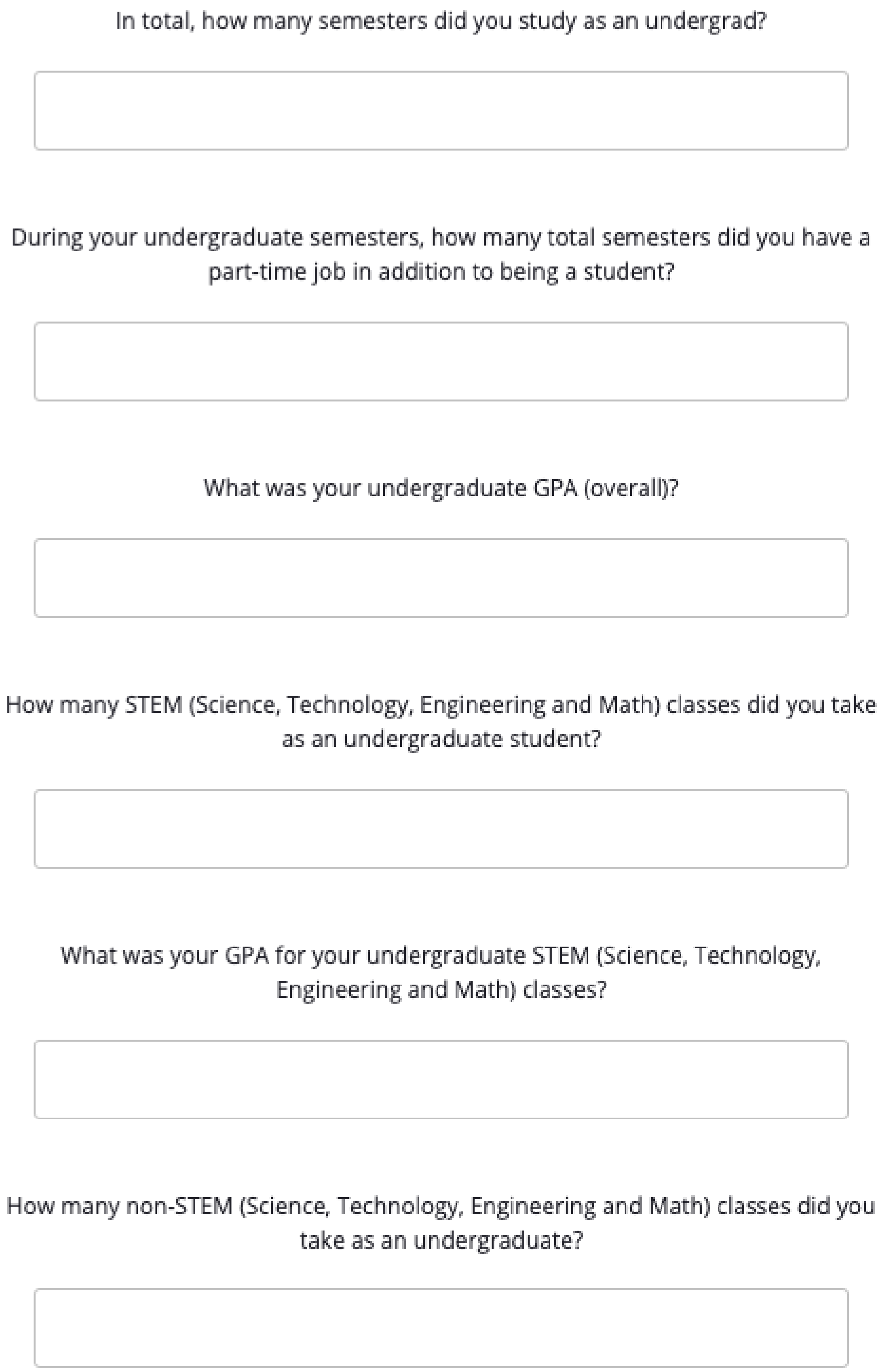
In total, how many semesters did you study as an undergrad?

During your undergraduate semesters, how many total semesters did you have a part-time job in addition to being a student?

What was your undergraduate GPA (overall)?

How many STEM (Science, Technology, Engineering and Math) classes did you take as an undergraduate student?

What was your GPA for your undergraduate STEM (Science, Technology, Engineering and Math) classes?

How many non-STEM (Science, Technology, Engineering and Math) classes did you take as an undergraduate?

**Supplementary Figure 16. Application Portal, Part 3**
Supplementary Figure 16 shows part 3 of the application in the website application portal.

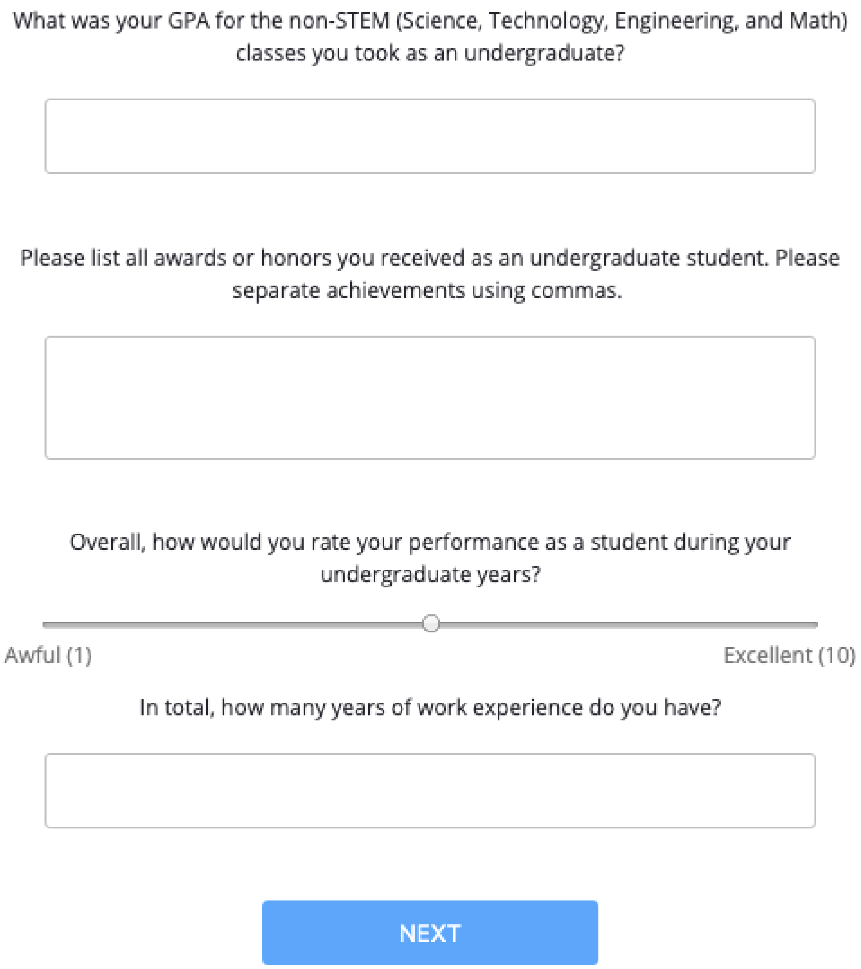
What was your GPA for the non-STEM (Science, Technology, Engineering, and Math) classes you took as an undergraduate?

Please list all awards or honors you received as an undergraduate student. Please separate achievements using commas.

Overall, how would you rate your performance as a student during your undergraduate years?

Awful (1)

Excellent (10)

In total, how many years of work experience do you have?

NEXT

**Supplementary Figure 17. Application Portal, Part 4**

Supplementary Figure 17 shows part 4 of the application in the website application portal.

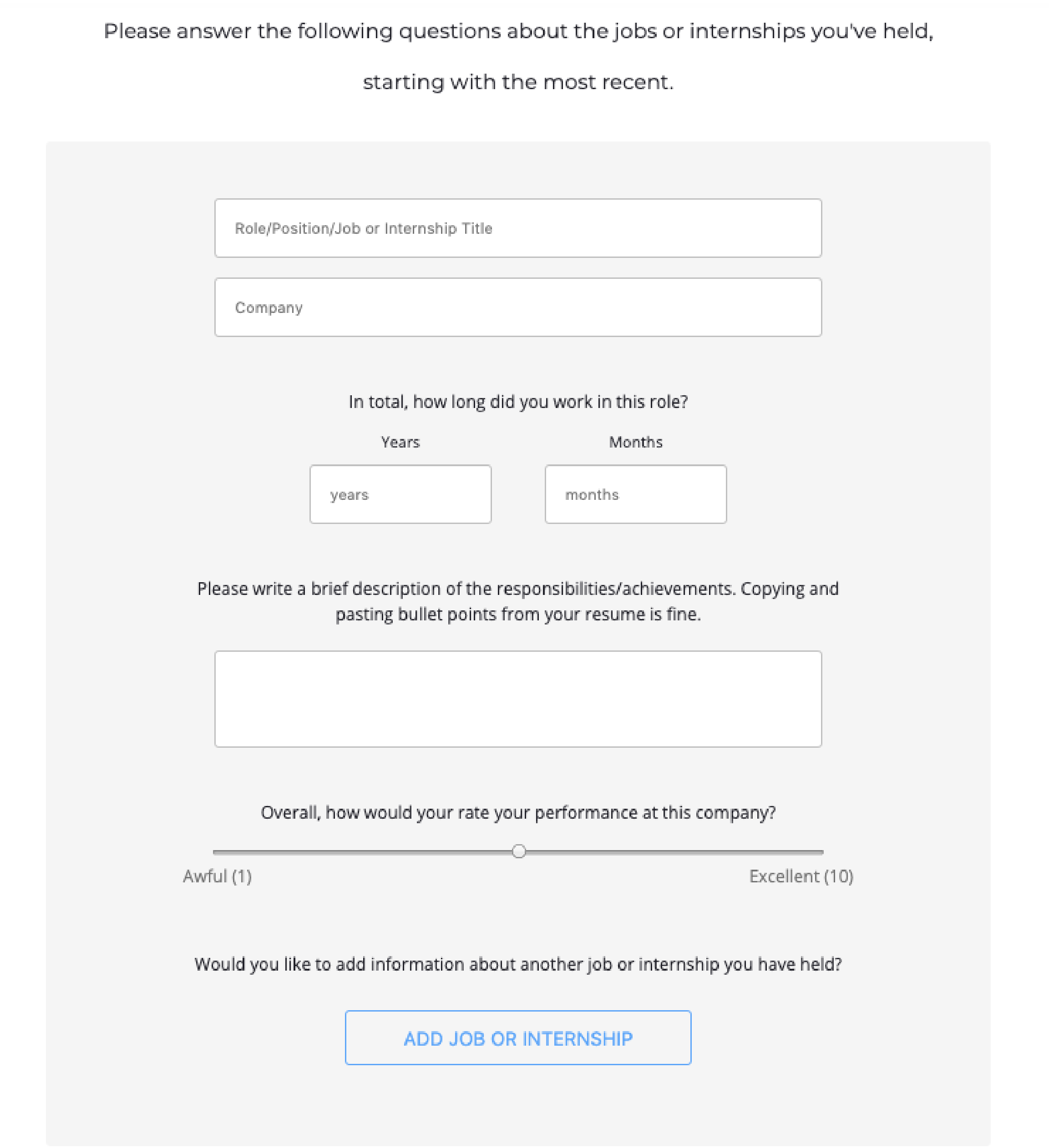

**Supplementary Figure 18. Application Portal, Part 5**

Supplementary Figure 18 shows part 5 of the application in the website application portal. Note, the skills displayed here are for the Full Stack Engineer application.

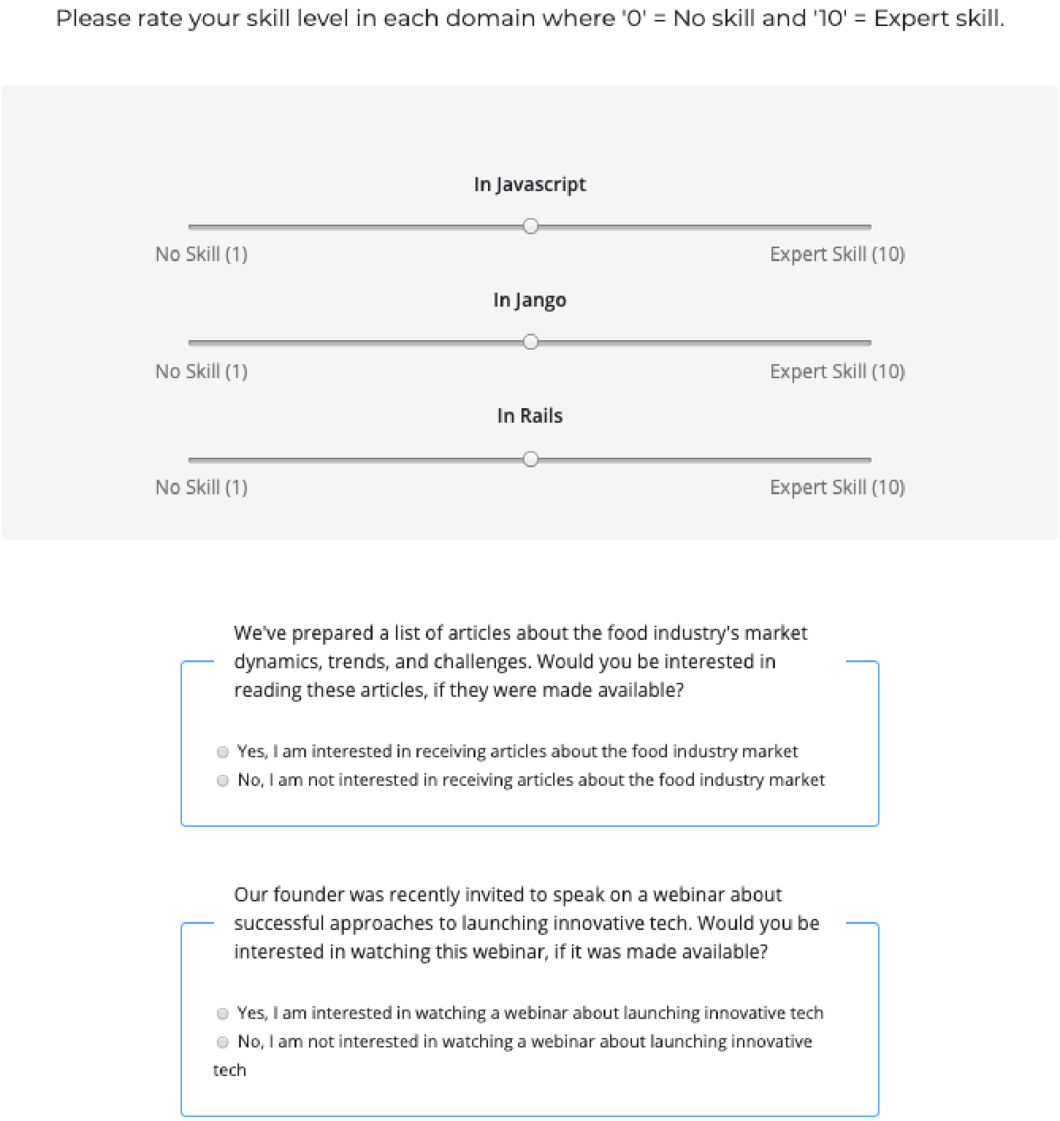

**Supplementary Figure 19. Application Portal, Part 6**

Supplementary Figure 19 shows part 6 of the application in the website application portal.

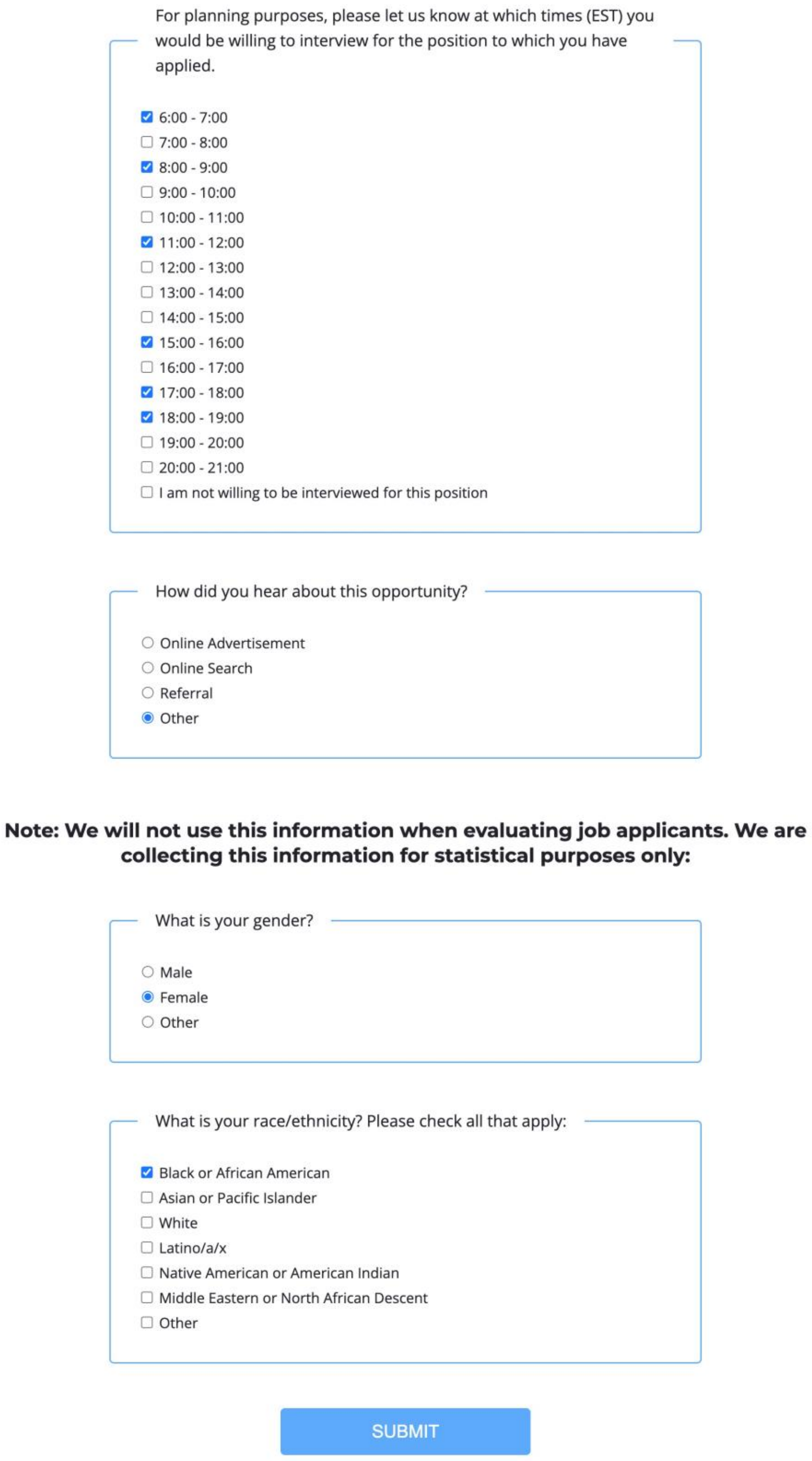

For planning purposes, please let us know at which times (EST) you would be willing to interview for the position to which you have applied.

- [x] 6:00 - 7:00
- [ ] 7:00 - 8:00
- [x] 8:00 - 9:00
- [ ] 9:00 - 10:00
- [ ] 10:00 - 11:00
- [x] 11:00 - 12:00
- [ ] 12:00 - 13:00
- [ ] 13:00 - 14:00
- [ ] 14:00 - 15:00
- [x] 15:00 - 16:00
- [ ] 16:00 - 17:00
- [x] 17:00 - 18:00
- [x] 18:00 - 19:00
- [ ] 19:00 - 20:00
- [ ] 20:00 - 21:00
- [ ] I am not willing to be interviewed for this position

How did you hear about this opportunity?

- ○ Online Advertisement
- ○ Online Search
- ○ Referral
- ◉ Other

**Note: We will not use this information when evaluating job applicants. We are collecting this information for statistical purposes only:**

What is your gender?

- ○ Male
- ◉ Female
- ○ Other

What is your race/ethnicity? Please check all that apply:

- [x] Black or African American
- [ ] Asian or Pacific Islander
- [ ] White
- [ ] Latino/a/x
- [ ] Native American or American Indian
- [ ] Middle Eastern or North African Descent
- [ ] Other

SUBMIT

**Supplementary Figure 20. Original Applicant Quality Rubric**
Supplementary Figure 20 shows the original applicant quality rubric. Due to overlapping criteria, the rubric was later amended (See 'Deviations' in Manuscript)

| Candidate: |
|---|
| |
| **Evaluator:** |
| |

| DATE |
|---|
| |

| RUBRIC | SCORE |
|---|---|
| Extremely Acceptable; Highest skill set; Extremely strong | **5** |
| Very Acceptable; High skill set; Very strong | **4** |
| Acceptable; Moderate strong skill set; Moderately strong | **3** |
| Slightly Acceptable; Guidelines somewhat met; Somewhat strong | **2** |
| Not Acceptable; Guidelines not met; Not at all strong | **1** |

| SCORING SCALE | TOTAL |
|---|---|
| **EXTREMELY ACCEPTABLE** | **5** |
| **VERY ACCEPTABLE** | **4** |
| **ACCEPTABLE** | **3** |
| **SLIGHTLY ACCEPTABLE** | **2** |
| **NOT ACCEPTABLE** | **1** |

| CRITERIA | 5 | 4 | 3 | 2 | 1 |
|---|---|---|---|---|---|
| **Interpersonal Skills:**<br>Does the candidate possess the required technical skills to perform the job OR have they demonstrated the ability to perform these skills? | | | | | |
| **Education/Knowledge:**<br>Does the candidate demonstrate strong work experience or internship? | | | | | |
| **Knowledge and Skills in Research:**<br>Does the candidate demonstrate clear understanding / with interested in webinars and receiving articles about the industry? | | | | | |
| **Leadership and Collegiality:**<br>Has experience in effectively working with others on various types of projects. | | | | | |

## FIGURE CREDIT LINES

**Figure 1:**
John Blackwell: baranq/stock.adobe.com
Alex Johnson: Rido/stock.adobe.com
Sam Smithson: sharplaninac/stock.adobe.com
Josh Goldstein: deagreez/stock.adobe.com
Dan Levitt (panel a): Africa Studio/stock.adobe.com
Jake Elfson: Rido/stock.adobe.com
Adam Stonefield: deagreez/stock.adobe.com
Alex Morgan (panel a): Zarya Maxim/stock.adobe.com
Blaine Smith (panel a): Rido/stock.adobe.com
James Livingston: MTomicic/stock.adobe.com
Oleg Ivanovich: contrastwerkstatt/stock.adobe.com
Brad Jackson: Minerva Studio/stock.adobe.com
Sam Gosford: Rido/stock.adobe.com
Michael Linmord: stokkete/stock.adobe.com
Justin Alborne: Syda Productions/stock.adobe.com
Jack Ibnone: Lisa F. Young/stock.adobe.com
Jose Garcia: David Gilder/stock.adobe.com
Dan Levitt (panel b): ArenaCreative/stock.adobe.com
Brian Nguyen: ajr_images/stock.adobe.com
Alex Morgan (panel b, d): Wayhome Studio/stock.adobe.com
Blaine Smith (panel b): michaeljung/stock.adobe.com
Pranav Gupta: and.one/stock.adobe.com
Farhad Karimi: Jacob Lund/stock.adobe.com
Jason Kim: WONG SZE FEI/stock.adobe.com
Josephine Garst: digitalskillet1/stock.adobe.com
Danielle Levitt: sepy/stock.adobe.com
Brianne Nost: iStock.com/Tempura
Alexa Morgan: Yuriy Shevtsov/ stock.adobe.com
Blaine Smith: (panel c, d): elnariz/stock.adobe.com
Ashley Worth: gpointstudio/stock.adobe.com
Gina Gregson (panel c): vgstudio/stock.adobe.com
Dawn Nokiwicz: pathdoc/stock.adobe.com
Danielle Cho: makistock/stock.adobe.com
Priya Gupta: arekmalang/stock.adobe.com
Gina Gregson (panel d): michaeljung/stock.adobe.com
Dawn Albertson: iStock.com/andresr

**FIGURE CREDIT LINES**

**Supplementary Figure 3:**
John Blackwell: baranq/stock.adobe.com
Alex Johnson: Rido/stock.adobe.com
Sam Smithson: sharplaninac/stock.adobe.com
Josh Goldstein: deagreez/stock.adobe.com
Dan Levitt (panel a): Africa Studio/stock.adobe.com
Jake Elfson: Rido/stock.adobe.com
Adam Stonefield: deagreez/stock.adobe.com
Alex Morgan (panel a): Zarya Maxim/stock.adobe.com
Blaine Smith (panel a): Rido/stock.adobe.com
James Livingston: MTomicic/stock.adobe.com
Oleg Ivanovich: contrastwerkstatt/stock.adobe.com
Brad Jackson: Minerva Studio/stock.adobe.com
Sam Gosford: Rido/stock.adobe.com
Michael Linmord: stokkete/stock.adobe.com
Justin Alborne: Syda Productions/stock.adobe.com
Jack Ibnone: Lisa F. Young/stock.adobe.com

**Supplementary Figure 4:**
John Blackwell: baranq/stock.adobe.com
Alex Johnson: Rido/stock.adobe.com
Sam Smithson: sharplaninac/stock.adobe.com
Josh Goldstein: deagreez/stock.adobe.com
Dan Levitt (panel a): Africa Studio/stock.adobe.com
Jake Elfson: Rido/stock.adobe.com
Adam Stonefield: deagreez/stock.adobe.com
Alex Morgan (panel a): Zarya Maxim/stock.adobe.com
Blaine Smith (panel a): Rido/stock.adobe.com
James Livingston: MTomicic/stock.adobe.com
Oleg Ivanovich: contrastwerkstatt/stock.adobe.com
Brad Jackson: Minerva Studio/stock.adobe.com
Sam Gosford: Rido/stock.adobe.com
Michael Linmord: stokkete/stock.adobe.com
Justin Alborne: Syda Productions/stock.adobe.com
Jack Ibnone: Lisa F. Young/stock.adobe.com
