# Supplementary material for: A field study of the impacts of workplace diversity on the recruitment of minority group members
Source: Nat Hum Behav. 2023 Oct 30;7(12):2212–27. doi: 10.1038/s41562-023-01731-5 (PMC10730395; doi:10.1038/s41562-023-01731-5)
Supplement: Supplementary file 2 — Reporting Summary [file 41562_2023_1731_MOESM2_ESM.pdf]

nature portfolio

Corresponding author(s): Nichols, Aaron; Axt, Jordan

Last updated by author(s): Jul 25, 2023

Reporting Summary

Nature Portfolio wishes to improve the reproducibility of the work that we publish. This form provides structure for consistency and transparency in reporting. For further information on Nature Portfolio policies, see our Editorial Policies and the Editorial Policy Checklist.

Statistics

For all statistical analyses, confirm that the following items are present in the figure legend, table legend, main text, or Methods section.

n/a	Confirmed	
☐	☒	The exact sample size (*n*) for each experimental group/condition, given as a discrete number and unit of measurement
☐	☒	A statement on whether measurements were taken from distinct samples or whether the same sample was measured repeatedly
☐	☒	The statistical test(s) used AND whether they are one- or two-sided<br>*Only common tests should be described solely by name; describe more complex techniques in the Methods section.*
☐	☒	A description of all covariates tested
☐	☒	A description of any assumptions or corrections, such as tests of normality and adjustment for multiple comparisons
☐	☒	A full description of the statistical parameters including central tendency (e.g. means) or other basic estimates (e.g. regression coefficient) AND variation (e.g. standard deviation) or associated estimates of uncertainty (e.g. confidence intervals)
☐	☒	For null hypothesis testing, the test statistic (e.g. *F*, *t*, *r*) with confidence intervals, effect sizes, degrees of freedom and *P* value noted<br>*Give P values as exact values whenever suitable.*
☒	☐	For Bayesian analysis, information on the choice of priors and Markov chain Monte Carlo settings
☒	☐	For hierarchical and complex designs, identification of the appropriate level for tests and full reporting of outcomes
☐	☒	Estimates of effect sizes (e.g. Cohen's *d*, Pearson's *r*), indicating how they were calculated

Our web collection on statistics for biologists contains articles on many of the points above.

Software and code

Policy information about availability of computer code

Data collection	Data were collected by javascript software on a custom-built survey. All website source code is available at osf.io/pf8am
Data analysis	R v 4.2.2, SPSS v27. Analysis and cleaning syntax are available at osf.io/vaq2g

For manuscripts utilizing custom algorithms or software that are central to the research but not yet described in published literature, software must be made available to editors and reviewers. We strongly encourage code deposition in a community repository (e.g. GitHub). See the Nature Portfolio guidelines for submitting code & software for further information.

Data

Policy information about availability of data

All manuscripts must include a data availability statement. This statement should provide the following information, where applicable:

- Accession codes, unique identifiers, or web links for publicly available datasets
- A description of any restrictions on data availability
- For clinical datasets or third party data, please ensure that the statement adheres to our policy

All data needed to recreate analyses are available at osf.io/vaq2g. See manuscript for full data availability statement.

Research involving human participants, their data, or biological material

Policy information about studies with human participants or human data. See also policy information about sex, gender (identity/presentation), and sexual orientation and race, ethnicity and racism.

Reporting on sex and gender	Participants reported gender using response options of "Male", "Female" and "Other". Analyses using participant gender used this self-report variable. Gender was considered in study design; one factor of our manipulation was the presence or absence of women employees in the company website. Of participants who submitted an application, 967 were male and 618 were female. Participant gender was included in analyses where possible (see Analyses 1-2). Consent for sharing individual-level data was not collected due to a consent waiver provided by ethic review.
Reporting on race, ethnicity, or other socially relevant groupings	Participants reported "race/ethnicity" using the following response options (participants could select multiple): "Black or African American", "Asian or Pacific Islander", "White", "Latino/a/x", "Native American or American Indian", "Middle Eastern or North African Descent", "Other". Analyses of race were done using this self-report variable. Response options were selected based on recommendations from the US Census. No control variables were included in analyses that considered participant race/ethnicity.
Population characteristics	See 'Behavioral & Social Sciences Study Design' section below.
Recruitment	Participants were recruited from posting job advertisements on LinkedIn between February 2021 - May 2021 and June 2021-June 2022. Recruitment was then limited to participants who have a LinkedIn profile and were interested in a job with a technology company.
Ethics oversight	Research protocol was approved by Duke University's Institutional Review Board for Social and Behavioral Sciences.

Note that full information on the approval of the study protocol must also be provided in the manuscript.

Field-specific reporting

Please select the one below that is the best fit for your research. If you are not sure, read the appropriate sections before making your selection.

☐ Life sciences ☒ Behavioural & social sciences ☐ Ecological, evolutionary & environmental sciences

For a reference copy of the document with all sections, see nature.com/documents/nr-reporting-summary-flat.pdf

Behavioural & social sciences study design

All studies must disclose on these points even when the disclosure is negative.

Study description	All data are quantitative and used an experimental design.
Research sample	Participants were recruited from advertisements from the website LinkedIn. Samples were not intended to be representative of any population. Study sample was selected based on interest in seeking employment at a technology company.
Sampling strategy	Sample was a convenience sample. Target sample sizes were determined in our Stage 1 protocol, and were selected because they provided 95% power for detecting an effect of theoretical and practical relevance. Details about power analysis calculations are available in our Stage 1 protocol.
Data collection	Data collection was done virtually via computer using the website source code, which randomly assigned participants to condition and tracked whether participants clicked on a job ad, started a job ad, and submitted an application. Self-report data was collected virtually using the website's custom-designed survey.
Timing	Data was collected in two waves: February 2021 - May 2021 and June 2021 - June 2022. Data collection was interrupted to allow for an additional ethics review in light of the Covid-19 pandemic.
Data exclusions	We excluded participants who 1) spent less than 15 seconds on the website (n = 121), 2) submitted clearly fake applications (n = 2), 3) indicated hearing about the position through a referral (n = 26), 4) reported their gender as "other" (n=22), and 5) bypassed the website and accessed the application survey directly (n=13). These criteria were not mutually exclusive, and in total 168 participants were removed from primary analyses.
Non-participation	Aside from the criteria outlined in the 'Data Exclusions' section, no participants were removed for non-participation. This is because 1) our protocol did not require consent, so participants could not opt out, and 2) Analysis 3 included all website visitors.
Randomization	Participants were randomly assigned to participants virtually, using the source code for the website.

Reporting for specific materials, systems and methods

We require information from authors about some types of materials, experimental systems and methods used in many studies. Here, indicate whether each material, system or method listed is relevant to your study. If you are not sure if a list item applies to your research, read the appropriate section before selecting a response.

Materials & experimental systems

n/a	Involved in the study
☒	☐ Antibodies
☒	☐ Eukaryotic cell lines
☒	☐ Palaeontology and archaeology
☒	☐ Animals and other organisms
☒	☐ Clinical data
☒	☐ Dual use research of concern
☒	☐ Plants

Methods

n/a	Involved in the study
☒	☐ ChIP-seq
☒	☐ Flow cytometry
☒	☐ MRI-based neuroimaging
